# Ethenoguanines Undergo Glycosylation by Nucleoside 2′-Deoxyribosyltransferases at Non-Natural Sites

**DOI:** 10.1371/journal.pone.0115082

**Published:** 2014-12-18

**Authors:** Wenjie Ye, Debamita Paul, Lina Gao, Jolita Seckute, Ramiah Sangaiah, Karupiah Jayaraj, Zhenfa Zhang, P. Alexandre Kaminski, Steven E. Ealick, Avram Gold, Louise M. Ball

**Affiliations:** 1 Department of Environmental Sciences and Engineering, Gillings School of Global Public Health, The University of North Carolina, Chapel Hill, Chapel Hill, North Carolina, United States of America; 2 Department of Chemistry and Chemical Biology, Cornell University, Ithaca, New York, United States of America; 3 Institut Pasteur, Unité de Chimie et Biocatalyse, UMR CNRS, Paris, France; Weizmann Institute of Science, Israel

## Abstract

Deoxyribosyl transferases and functionally related purine nucleoside phosphorylases are used extensively for synthesis of non-natural deoxynucleosides as pharmaceuticals or standards for characterizing and quantitating DNA adducts. Hence exploring the conformational tolerance of the active sites of these enzymes is of considerable practical interest. We have determined the crystal structure at 2.1 Å resolution of *Lactobacillus helveticus* purine deoxyribosyl transferase (PDT) with the tricyclic purine 8,9-dihydro-9-oxoimidazo[2,1-*b*]purine (*N* ^2^,3-ethenoguanine) at the active site. The active site electron density map was compatible with four orientations, two consistent with sites for deoxyribosylation and two appearing to be unproductive. In accord with the crystal structure, *Lactobacillus helveticus* PDT glycosylates the 8,9-dihydro-9-oxoimidazo[2,1-*b*]purine at N7 and N1, with a marked preference for N7. The activity of *Lactobacillus helveticus* PDT was compared with that of the nucleoside 2′-deoxyribosyltransferase enzymes (DRT Type II) from *Lactobacillus leichmannii* and *Lactobacillus fermentum*, which were somewhat more effective in the deoxyribosylation than *Lactobacillus helveticus* PDT, glycosylating the substrate with product profiles dependent on the pH of the incubation. The purine nucleoside phosphorylase of *Escherichia coli*, also commonly used in ribosylation of non-natural bases, was an order of magnitude less efficient than the transferase enzymes. Modeling based on published active-site structures as templates suggests that in all cases, an active site Phe is critical in orienting the molecular plane of the purine derivative. Adventitious hydrogen bonding with additional active site residues appears to result in presentation of multiple nucleophilic sites on the periphery of the acceptor base for ribosylation to give a distribution of nucleosides. Chemical glycosylation of *O* ^9^-benzylated 8,9-dihydro-9-oxoimidazo[2,1-*b*]purine also resulted in N7 and N1 ribosylation. Absent from the enzymatic and chemical glycosylations is the natural pattern of N3 ribosylation, verified by comparison of spectroscopic and chromatographic properties with an authentic standard synthesized by an unambiguous route.

## Introduction

Non-natural deoxynucleosides and deoxynucleoside analogs are important as therapeutic drugs [1]–[3], as probes for mechanisms of parasite-transmitted disease [4] and mechanisms of DNA repair [5] and for identifying and characterizing DNA damage. Chemical synthesis of non-natural nucleosides typically involves protection/deprotection steps and reaction conditions under which the glycosidic bond may be labile, resulting in low yields and difficult purification. Enzymic deoxyribosylation of modified nucleobases or glycosylation with non-natural sugars can offer an alternative synthetic pathway with high yields and stereo- and regioselectivity. As a consequence, the synthetic utility of deoxyribosyltransferase (DRT) and purine nucleoside phosphorylase (PNP) enzymes has been explored. Two classes of DRT enzymes can be isolated from the Lactobacilli species *Lactobacillus helveticus* (*L. helveticus*) and *Lactobacillus leichmanii* (*L. leichmanii*) [6]. Type I DRT (purine deoxyribosyltransferase; PDT) enzymes transfer deoxyribose groups exclusively from purine to purine, while Type II DRT (nucleoside deoxyribosyltransferase; NDT) enzymes can utilize purines and pyrimidines as both donors and acceptors. Although the PDT and NDT enzymes show some structural similarity [6], NDT enzymes have been favored as biocatalysts since they are more flexible than PDT enzymes with regard to the type of donor base while retaining absolute stereospecificity for generating the β-deoxyribose anomer [3], thus expanding the pool of available donors and acceptors for transfer of modified sugars. *Escherichia coli* (*E. coli*) PNP in the presence of uridine or thymidine phosphorylases and the appropriate deoxyribose donor has also been used for this purpose [1], [2], [7].

**Figure 1:**
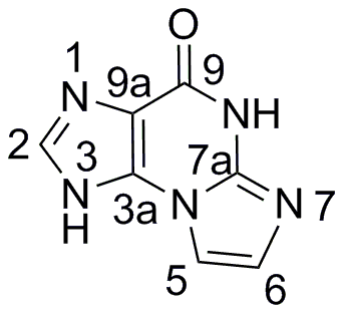
Structure and numbering convention of 8,9-dihydro-9-oxoimidazo[2,1-*b*]purine (1).

To define the range of structures suitable as deoxyribosyl acceptors, a number of structural studies of DRTs and PNPs have been undertaken [8]–[10]. Although deoxyribosyl transfer to purine or pyrimidine acceptors is highly regioselective for natural substrates, modified bases or base analogs may be deoxyribosylated at multiple sites [8], [11]–[14]. *L. helveticus* PDT transfers 2-deoxyribose to N3, N7 or N9 of sterically compact guanine derivatives [8], suggested by structural studies to be a consequence of latitude in substrate orientation within the active site through alternative hydrogen bonding schemes with active-site residues. Here we investigate deoxyribosylation of the angular tricyclic base 8,9-dihydro-9-oxoimidazo[2,1-*b*]purine (**1**; Fig. 1) to determine active site steric constraints of DRT enzymes with a sterically demanding acceptor.

The tricyclic framework formed by fusion of a 5-membered ring on the Watson-Crick pairing edge of the nucleobase between the exocyclic *N* ^2^ and endocyclic N3 of guanine renders **1** sterically bulkier than guanine analogs previously investigated by X-ray crystallography. In a study of product distributions from glycosylation of a series of substrates, a crude PDT preparation isolated from *L. helveticus* has been reported to deoxyribosylate **1** at N1 and N3 [14]. We report the structure of **1** complexed at the active site of *L. helveticus* PDT and take advantage of available crystal structures of the NDT from *L. leichmannii* and of the *E. coli* PNP to examine more generally the regiochemistry of the enzymatic glycosylation by modeling **1** at the active sites of these enzymes. We have generated product profiles from transdeoxyribosylation *in vitro* by *L. helveticus* PDT, the *L. leichmannii* and *Lactobacillus fermentum* NDTs, and with *E. coli* PNP and discuss the product profiles generated from the enzymatic deoxyribosylation in terms of the crystal structure and modeling results. For comparison, we have also determined the deoxyribosylation products obtained from **1** by a published chemical reaction.

## Materials and Methods

### Chemicals

Solvents were HPLC grade and were purchased from Fisher Scientific Co. or Mallinckrodt Baker, Inc., except for ethanol, which was purchased from AAPER Alcohol and Chemical Co. Ammonium hydroxide, sodium bicarbonate, HCl, acetic acid, acetic anhydride, and potassium monohydrogen phosphate were obtained from Fisher Scientific Co. 2′-Deoxyguanosine was purchased from USB Corp. and benzyl alcohol from J. T. Baker. All other reagents were purchased from Sigma-Aldrich and used as received. Hydrogen gas was purchased from National Welders Supply Co. 3,5-Di-*O*-(*p*-toluyl)-2-deoxy-D-ribofuranosyl chloride was synthesized according to a published procedure [15], as was *O* ^6^-benzylguanine [16]. 8,9-Dihydro-9-oxoimidazo[2,1-*b*]purine (**1**) was synthesized [17] and glycosylated [18], [19] according to published procedures, described in detail, along with complete characterization, as S1 Materials. By ^1^H NMR and HPLC analysis, the final product was greater than 95% pure, with 5,9-dihydro-9-oxoimidazo[1,2-*a*]purine (**2**) as an identifiable impurity. 8,9-Dihydro-9-oxo-3-(2-deoxy-β-D-ribofuranosyl)-imidazo[2,1-*b*]purine (**3**) was synthesized as a reference standard by a published procedure [20], described in detail in S1 Materials, along with definitive characterization by 2D NMR methods.

### Instrumentation

NMR spectra were recorded on a Varian Inova NMR spectrometer equipped with a cold probe at 500 MHz for acquisition of ^1^H data and 125 MHz for ^13^C data. Low resolution ESI-MS/MS data were acquired on a Finnigan DECA system. High resolution mass measurements were obtained on a Bruker FT-ICR-MS equipped with a capillary ESI source by flow injection of 4–6 µL samples, with angiotensin I (0.02 mg/mL) as the calibration standard. UV-vis spectra were recorded on a Cary 300, with Cary Win UV software. HPLC was carried out on a Thermo LC with an Altech UV-Vis detector and Ezstar software (EZCHROM).

### Chromatography

Both analytical and semi-preparative HPLC separations were carried out on a reverse phase Eclipse XDB C18 column (150×4.6 mm) at a flow rate of 1 mL/min, as described below. Analytical thin-layer chromatography (TLC) was performed on silica-coated aluminum plates (particle size 17 µm, 200 µm thickness) purchased from Sigma-Aldrich, and preparative TLC on silica-coated glass plates (particle size 40-63 µm, 500 or 1000 µm thickness), purchased from Analtech Inc.

### Enzymes

*L. helveticus* PDT, *L. fermentum* NDT [21] and *L. leichmannii* NDT were purified as follows. 500 mL of LB medium inoculated with an overnight culture of BL21(DE3)pLysS containing either pETLH4 (*L. helveticus* PTD), pETLL7 (*L. leichmannii* NDT), or pLF6 (*L. fermentum* NDT) was grown under agitation at 37°C until A_600_≈0.6. Isopropyl-1-thio-β-D-galacto-pyranoside was added to a final concentration of 1 mM, and the cultures were incubated for 2.5 h. Bacteria were centrifuged, washed once with 0.1 M phosphate buffer (pH 7.5). Pellets were frozen at −20°C. Cells were resuspended in 20 mL of phosphate buffer and broken by one passage through a French press at 14000 p.s.i. The lysate was centrifuged at 23,000×g for 1 h, and the supernatant was precipitated by addition of solid ammonium sulfate to 30–40% saturation. Proteins were pelleted by centrifugation at 8,000×g for 30 min and resuspended in phosphate buffer. Each protein was further purified by filtration on a Sephacryl S-200 column previously equilibrated in sodium phosphate buffer containing 0.1 M NaCl (pH 6.0). The elution was followed by UV absorption at 280 nm, and each fraction was analyzed by SDS-PAGE electrophoresis and by following the transfer activity using dC+A for the NDTs and dG+ A for the PDT. Protein concentrations were measured by UV absorption. PNP (EC 2.4.2.1) and thymidine phosphorylase (EC 2.4.2.4) from *E. coli* were purchased from Sigma-Aldrich and used as received.

### PDT Crystallization Conditions

Pure protein was buffer exchanged into 20 mM 2-(*N*-morpholino)ethanesulfonic acid (pH 8.0) to a final concentration of 20 mg/mL. Native PDT crystals were grown at 22°C by the hanging-drop vapor diffusion method over 3–5 days. Drops containing 1 µL of protein and 1 µL of reservoir solution were found to be optimal for crystal growth. Diffraction quality crystals were obtained under the previously-optimized condition of 100 mM Tris (pH 7.9) and 2.2 M ammonium sulfate [8]. The native crystals were gradually soaked into stabilizing solutions of mother liquor containing 20–25% PEG 4000 and **1** from 2 mM up to 6 mM. The crystals were soaked in each solution for approximately one hour and kept overnight in the final solution at 6 mM **1**.

### Data Collection and Processing

The PDT-**1** complex datasets were collected at NE-CAT 24-ID-E beamline, at the Advanced Photon Source (Argonne National Laboratory, Argonne, IL) using a Quantum 315 detector (Area Detector Systems Corp.). Crystals were flash frozen in liquid nitrogen with 20% glycerol as cryoprotectant. The data were indexed, integrated and scaled using the HKL2000 program suite [22]. Data collection and data processing statistics are shown in Table 1.

**Table 1:** Summary of data collection statistics for PDT crystallized with 8,9-dihydro-9-oxoimidazo[2,1-*b*]purine^a^.

Parameters	Values
Resolution (Å)	2.1
Space group	P4^3^2^1^2
a, b (Å)	79.69
c (Å)	186.69
N/ASU	3
Matthews number	2.53
Solvent content (%)	55
Unique reflections	35578
Redundancy	5.9 (5.3)
Completeness	98.7 (93.6)
R_sym_ b (%)	4.6 (26.6)
I/σ	23.1 (4.8)

a Values for the highest resolution shell are given in parentheses.

b *R*_sym_ = ΣΣ_i_ I_i_−<I> |/Σ<I>, where <I> is the mean intensity of the N reflections with intensities I_i_ and common indices *h,k,l*.

### Model Building and Refinement

PDT crystallizes in the tetragonal space group P4_3_2_1_2 with 3 monomers in the asymmetric unit. The corresponding Matthews coefficient and solvent contents are 2.53 and 55% respectively [23]. The native PDT structure (PDB ID 1S2L) [8] was used as a starting model and the model was subsequently refined using rounds of rigid body refinement, simulated annealing, temperature factor refinement and minimization. Initial refinement cycles were performed with noncrystallographic symmetry (NCS) [24] and final rounds with the PHENIX suite of programs [25]. Coot [26] was used for model building. The NCS constraints were kept tight in the initial rounds of refinement and slowly relaxed in the final round. Water molecules were added and refined in the final rounds of refinement. The parameter and topology files for the ligand were generated with the Dundee PRODRG2 server [27]. The refinement statistics are summarized in Table 2. Complete structure factor data and final coordinates were deposited in the Protein Data Bank (www.rcsb.org): PDB ID code 4MEJ.

**Table 2:** Final refinement statistics for PDT crystallized with 8,9-dihydro-9-oxoimidazo[2,1-*b*]purine.

Parameters		Values	
Resolution (Å)		2.1	
Number of protein atoms		3844	
Number of water molecules		300	
Number of ligand atoms		44	
Root mean square deviation from ideal geometry			
bonds (Å)		0.004	
angles (deg)		0.852	
*R* factor^a^ (%)		19.52	
*R*_free_ b (%)		22.9	
Ramachandran plot			
most favored region (%)		86.8	
additionally allowed regions (%)		12.5	
generously allowed regions (%)		0.2	
disallowed regions (%)		0.5	

a *R* factor  = *Σ*_hkl_||F_obs_ |−k|F_cal_||/*Σ*_hkl_|F_obs_| where F_obs_ and F_cal_ are observed and calculated structure factors, respectively.

b For *R*_free_ the sum is extended over a subset of reflections (5%) excluded from all stages of refinement.

### Modeling of the active site of *L. leichmannii* NDT and *E. coli* PNP

Computational docking studies were based on docking of **1** into the active site cavities using AutoDock Vina 1.1.1 [28] followed by conformational searching for optimal orientations from docking to more rigorously explore the active site using Schrodinger MacroModel 9.9 [29]. For *L. leichmannii* NDT, PDB structure 1F8Y [9] with bound 5-methyl-2′-deoxypseudouridine (5-Me-dψUrd; 2.4 Å resolution) was used as a template, and for *E. coli* PNP, the template was PDB structure 1PK9 [10] with bound 2-fluoroadenosine (1.9 Å resolution). Phosphate and protonated Asp 204 were retained during the calculation. Compound **1** in its neutral form was subjected to the MacroModel 9.5.212 [30] minimization using OPLS 2005 (Optimized Potentials for Liquid Simulations) force field with water solvation treatment and a convergence threshold gradient of 0.01 [31]. Ligand diameter midpoint was set to a box of 6×6×6 Å encompassing the active site for receptor grid generation. No ligand constraints were set.

### Enzymatic Glycosylation

Enzymatic glycosylations were conducted under the following general conditions. Compound **1** (4.2 µmol) and deoxynucleoside donor (12.5 µmol) were dissolved in 0.1 M phosphate buffer adjusted to pH 7.5 with 1 M HCl or 0.5 M 2-[*N*-morpholino]ethanesulfonic acid buffer adjusted to pH 8.0 with 10 M NaOH and the transglycosylase was added to a final reaction volume of 10 mL. Reactions were incubated overnight at 45°C. For incubations with *L. helveticus* PDT, 40 µg enzyme were added with dGuo as donor, with *L. fermentum* and *L. leichmannii* NDTs, 40 µg enzyme were added with dCyd as donor. For glycosylation with thymidine phosphorylase (*E. coli*)/nucleoside phosphorylase, 0.846 IU phosphorylase and 2.536 unit PNP were incubated overnight with 25.5 µmol **1** and dThyd in 10 mL phosphate buffer (pH 8.0) at 41°C.

### HPLC Analysis and Isolation of Enzymatic Glycosylation Products

Incubations were filtered, lyophilized, redissolved in ∼2 mL H_2_O and the products separated by HPLC on an Eclipse XDB C18 column (100×4.6 mm) eluted at 1 mL/min, with a gradient from 5% methanol in 1 mM phosphate buffer (pH 8.0) to 12% methanol in 1 mM phosphate buffer over 20 min. The mixture from the thymidine phosphorylase/nucleoside phosphorylase incubation was filtered, reduced ∼50% in volume by lyophilization and then separated by HPLC as described above. Products were collected at 12, 16, 18 and 22 min (S1 Figure). For reference, authentic **3** eluted at 5.4 min in this system.

#### 12 Min fraction

UV-vis (H_2_O): λ_max_ (*ε*) 218 (22342), 263 (11056) nm. Exact mass (as the K^+^ adduct), *m/z* calc for C_12_H_13_N_5_O_4_K^+^ 330.0599, found 330.0599. ^1^H NMR (500 MHz, DMSO-*d_6_*) was identical to 8,9-dihydro-9-oxo-7-(*β*-D-2-deoxyribofuranosyl)-imidazo[2,1-*b*]purine from chemical glycosylation. (Complete NMR data are given in S1 Materials and S2 and S3 Figures).

#### 16 Min fraction

UV-vis (H_2_O): λ_max_ (*ε*) 217 (20867), 263 (9066) nm. Exact mass, *m/z* calc 292.1040, found 292.1039; (as K^+^ adduct) calc for C_12_H_13_N_5_O_4_K^+^ 330.0599, found 330.0599. ^1^H NMR (500 MHz, DMSO-*d_6_*) was identical to 8,9-dihydro-9-oxo-1-(*β*-D-2-deoxyribofuranosyl)-imidazo[2,1-*b*]purine from chemical glycosylation. (Complete NMR data are given in S1 Materials and S4 and S5 Figures).

#### 18 Min fraction

UV-vis (H_2_O): λmax (ε) 226 (15863), 284 (5318) nm. Exact mass (K^+^ adduct), m/z calc for C_12_H_13_N_5_O_4_K^+^ 330.0599, found 330.0598. (Complete NMR data are given in S1 Materials and S6 and S7 Figures).

#### 22 Min fraction

UV-vis (H_2_O): λ_max_ 228, 306 nm. Positive ESI-MS: m/z 330 [M+K]^+^, 292 [M+H]^+^, 176 [M+H–deoxyribose]^+^. (^1^H NMR data are given in S1 Materials and S8 Figure).

## Results

### Crystal Structure and Modeling of the *L. helveticus* PDT-1 Complex

**Figure 2:**
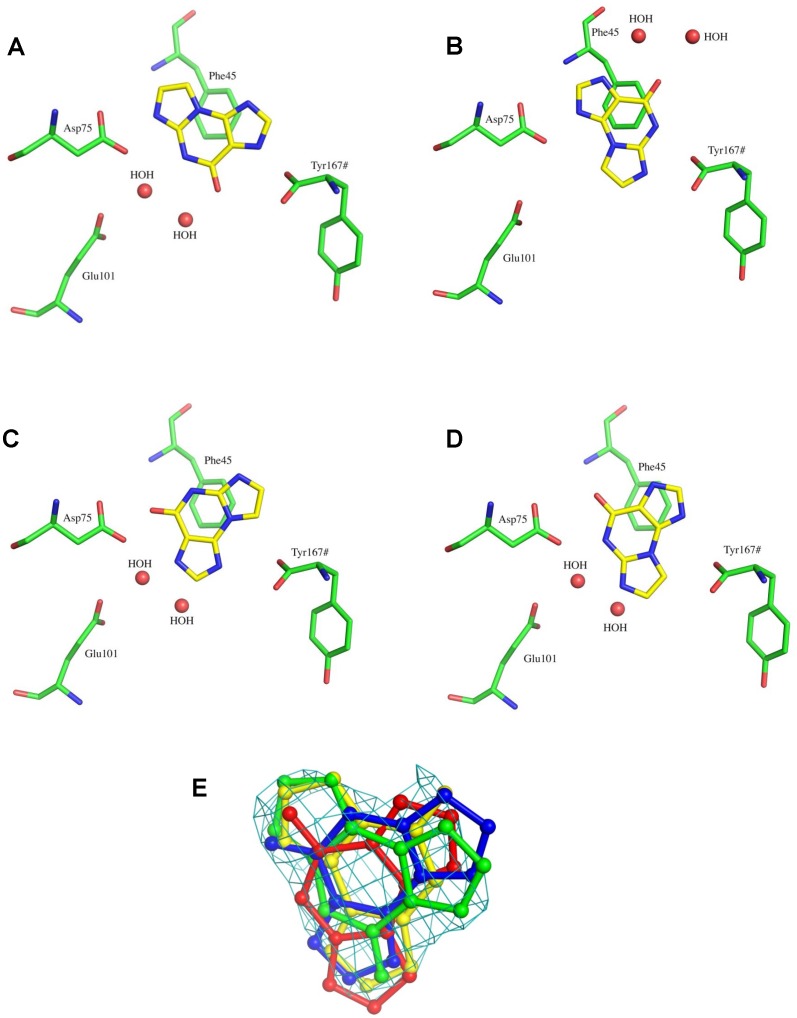
The active site of the *L. helveticus* PDT-1 complex, in the first (A), second (B), third (C) and fourth (D) conformations. The important surrounding residues are shown in stick representation. The protein C atoms are colored in green, N in blue and O in red. The ligand C atoms are colored yellow, N in blue and O in red. Graphics were generated from the crystal structures with PyMOL [32]. (E) The F_o_-F_c_ density for ethenoguanine in the active site of PDT contoured at 2.5 σ. The ligand, shown in ball and stick representation, is colored green, yellow, red and blue for the conformations respectively.

The initial F_o_-F_c_ electron density map revealed significant electron density for **1** in the active site of PDT at a contour level of 3 σ. However the density did not uniquely define the orientation of **1**, indicating the possibility of multiple conformations. Examination of the electron density suggests that the substrate binds in at least four different, overlapping orientations. In the first orientation (Fig. 2A), the base is anchored in the active site by four hydrogen bonding interactions. N7 forms a hydrogen bond with carboxyl oxygen OD1 or OD2 of Asp75, at an N–O bond distance of 2.6 Å or 2.9 Å, respectively. Another hydrogen bond can link N1 and a C-terminal oxygen atom of Tyr167# from an adjacent monomer (2.3 Å), while N8 is hydrogen bonded to a water molecule at the active site. The aromatic ring of the base is also stabilized by a π-stacking herringbone interaction with Phe45. The second conformation (Fig. 2B) suggests a binding mode in which the base is rotated by approximately −120° around an axis perpendicular to the plane of the imidazopurine. In this conformation N7 and N8 form hydrogen bonds with the C terminal carboxylate oxygen atoms of Tyr167# from the adjacent monomer, each with a bond distance of 2.7 Å. N3 is hydrogen bonded to Asp75 with a bond distance of 2.5 Å; N1 and *O* ^9^ participate in two hydrogen bonds with the active site water molecules. In the third conformation (Fig. 2C), *O* ^9^ is hydrogen-bonded to the carboxylate of Asp75 at a distance of 2.5 Å. N1 is within hydrogen bonding distance (2.9 Å) of Asp75 OD1, and N1 and N3 are hydrogen bonded to active site water molecules. In the fourth binding mode (Fig. 2D), N8 forms a hydrogen bond with OD1 of Asp75 at a distance of 2.4 Å. N3 makes a hydrogen bond with a distance of 2.7 Å to the C-terminal carboxylate of Tyr167# from the adjacent monomer and N7 forms a hydrogen bond with Glu101 via a water molecule. The π-stacking herringbone interaction with Phe45 is conserved in all four orientations. As discussed below, such π-stacking of an equivalent Phe with bound purines or purine analogs is preserved in NDT and PNP structures.

Refinements of the models revealed that none of the orientations individually accounts for the total electron density; a combination of the four orientations is required to fit the complete electron density (Fig. 2E). The occupancy and B values of the ligand were refined in each conformation. The B values were lower for the model in which all four of the conformations were included, compared to models where each conformation had a full occupancy; however, the level of resolution was not sufficient to determine which conformations were predominant, since all four were weighted equally in the model. Hence product profiles from enzymic glycosylations need to be examined in order to determine which of these configurations resulted in product formation, and their relative efficiency.

### Modeling of 1 Complexed with *L. leichmannii* NDT and *E. coli* PNP

**Figure 3:**
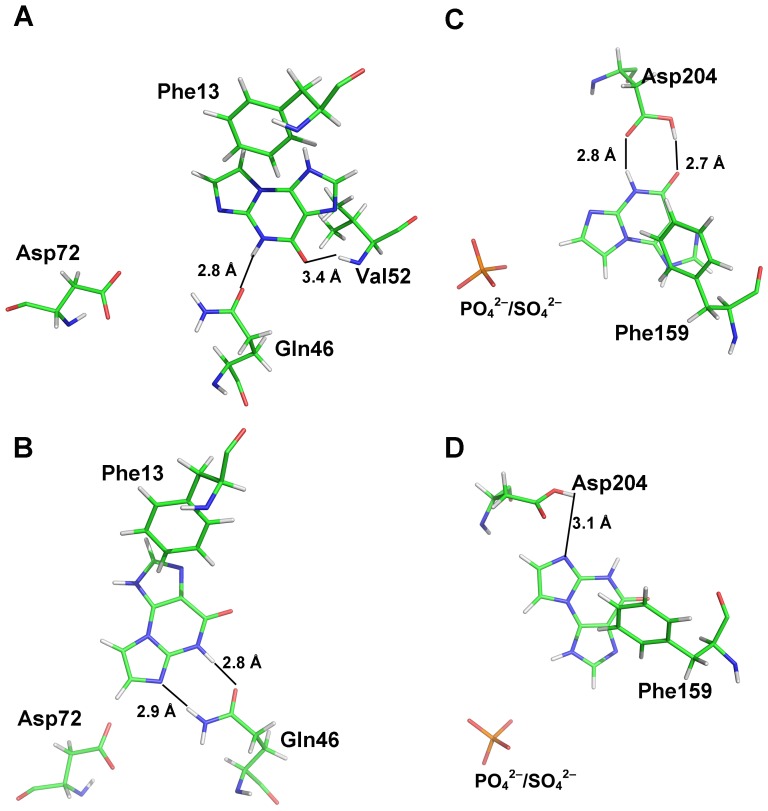
Active site model of *L. leichmannii* NDT-1 complex (A, B) and *E. coli* PNP-1 complex (C, D) showing the two most energetically favorable orientations in each case. CPK colors are used in the models. Hydrogen bonds are shown in green.

The model of **1** in the active site of *L. leichmannii* NDT yields two energetically favorable orientations (Fig. 3A, B). As in the case of the published structures with 5-Me-dψUrd and 3′-deoxyadenosine complexed at the active site [9], the molecular plane of the base is positioned by a π-stacking interaction with Phe13, which plays the same role as Phe45 in *L. helveticus* PDT and situates **1** virtually coplanar with 5-Me-dψUrd in the published structure. While in the case of 5-Me-dψUrd and 3′-deoxyadenosine active site residues Gln46 and Asp 72 are responsible for substrate binding in the plane of the base, only Gln46 anchors **1**. In the lowest energy orientation (Fig. 3A), H8 of **1** is hydrogen bonded to the carbonyl oxygen of Gln46. In this orientation, **1** is positioned to accept the deoxyribose at N7. A second low-energy orientation, related by a 60^o^ rotation in the molecular plane and displacement by ∼−1.5 Å along the molecular x-axis of the base (Fig. 3B), positions N1 to be deoxyribosylated. In this orientation, the base is anchored by hydrogen bonds between an amido hydrogen of Gln46 and N7 of **1** and between the carbonyl oxygen of Gln46 and NH8.

In *E. coli* PNP, the plane of **1** is positioned by a π-stacking interaction with Phe159, in the same manner as for purines in the PDT and NDT structures [10]. The active site residue responsible for positioning the molecular plane of the base with respect to rotation about the perpendicular axis is Asp204, as is the case for other purine derivatives (Fig. 3C, D). In the lowest energy configuration, the carboxy group of Asp204 is hydrogen bonded to N8H and *O* ^9^ of **1**, positioning N7 as acceptor of the deoxyribosyl group (Fig. 3C). In a second less energetically favorable orientation, the base is rotated −90^o^ in the molecular plane, so that the carboxy group of Asp204 now makes a single hydrogen bond to N7, and N1 is positioned to accept the deoxyribosyl group (Fig. 3D).

### Enzymatic Glycosylations

**Figure 4:**
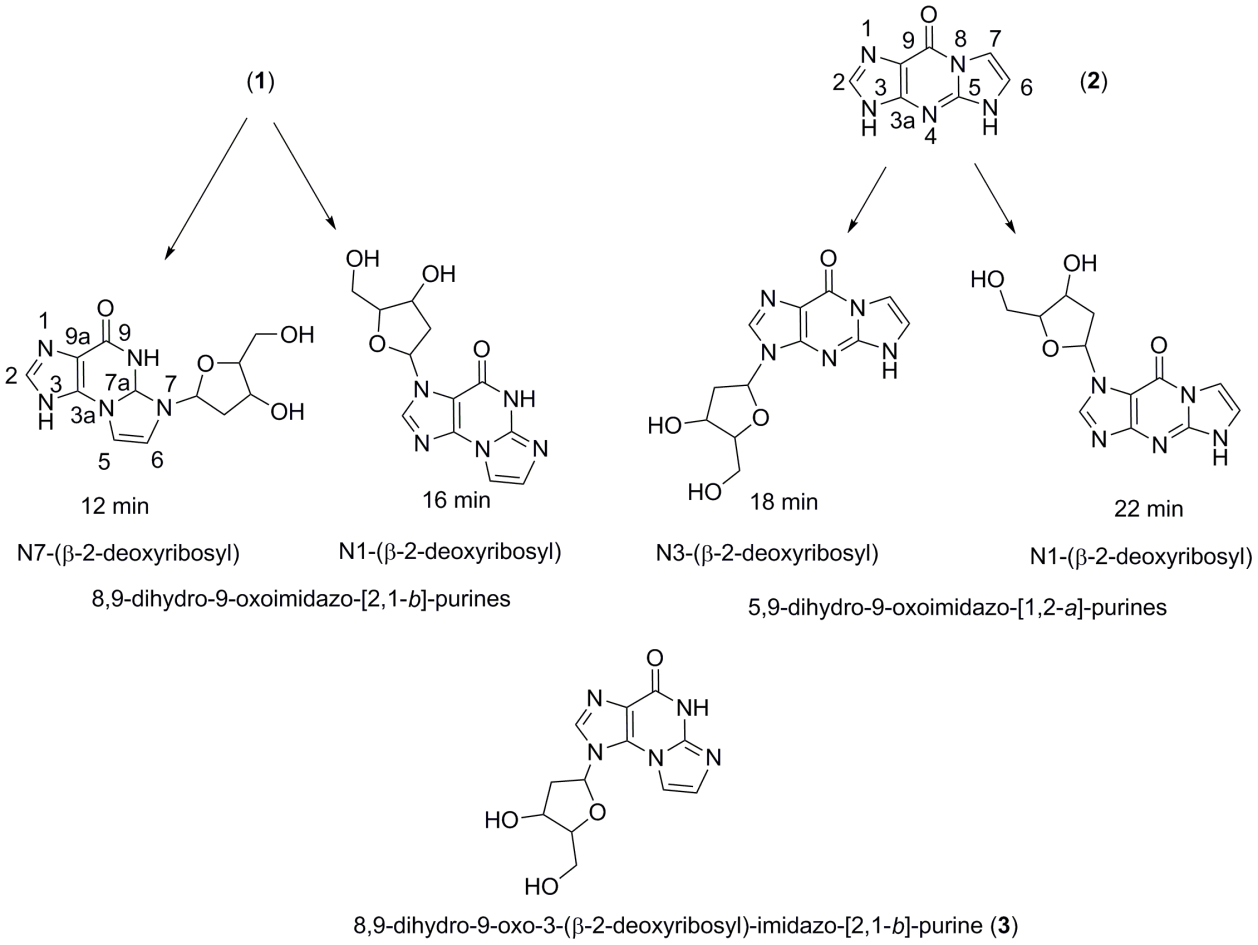
Structure and numbering conventions of the glycosylated oxoimidazopurine derivatives.

We investigated glycosylation of **1** by purified Type I (PDT) *trans N*-deoxyribosyltransferase from *L. helveticus*, the Type II transferases (NDT) from *L. leichmannii* (structurally similar to the transferase from *L. helveticus* [8]) and from *L. fermentum*, and by commercially available *E. coli* PNP. Glycosylations with the *trans N*-deoxyribosyltransferase enzymes were performed at pH 7.5 and 8.0 based on the reported steep pH-dependent activity of purified *L. leichmannii* [33; *J. Biol. Chem.* **1963**, *238*, 702], while the glycosylation with *E. coli* PNP was run under optimal conditions according to the published procedure [14]. The *Lactobacillus trans N*-deoxyribosyltransferase enzymes and *E. coli* PNP generated 2 major products with retention times of ∼12 and 16 min, having a major long-wavelength UV absorbance band at 260 nm as expected for the angularly-fused imidazo[2,1-*b*]purine chromophore. Two minor products with retention times of 18 and 22 min, representing no more than 4% of the substrate, were characterized by a broad, long wavelength UV band near 300 nm in the electronic spectra, characteristic of the linear etheno ring fusion of the imidazo[1,2-*a*]purine framework. As described below, the major products eluting at 12 and 16 min have been established as isomeric deoxynucleosides of **1**, while the minor products eluting at 18 and 22 min originated, as expected, from glycosylation of **2** (Fig. 4), present at a level of ∼4% in the substrate. The mass spectra of all products isolated from the enzymatic glycosylations correspond to addition of a deoxyribosyl moiety to a dihydro oximidazopurine framework. In ^1^H NMR spectra of imidazo[2,1-*b*]purines, the chemical shifts of the protons of the 5-membered fused (etheno) ring are strongly dependent on the solvent environment [34] and thus do not provide definitive structural identification. As a consequence, we confirmed the molecular structures of the enzymatic ribosylation products eluting at 12, 16 and 18 min by heteronuclear multiple bond shift correlation (HMBC) and nuclear Overhauser effect spectroscopy (NOESY) NMR. A sufficient quantity of the peak eluting at 22 min could not be collected for complete characterization by 2-dimensional NMR spectrometry and identification is therefore tentative. Expanded regions of the HMBC and NOESY spectra critical to structural determination are discussed in the text; complete NMR spectra are presented in S6 and S7 Figures.

**Figure 5:**
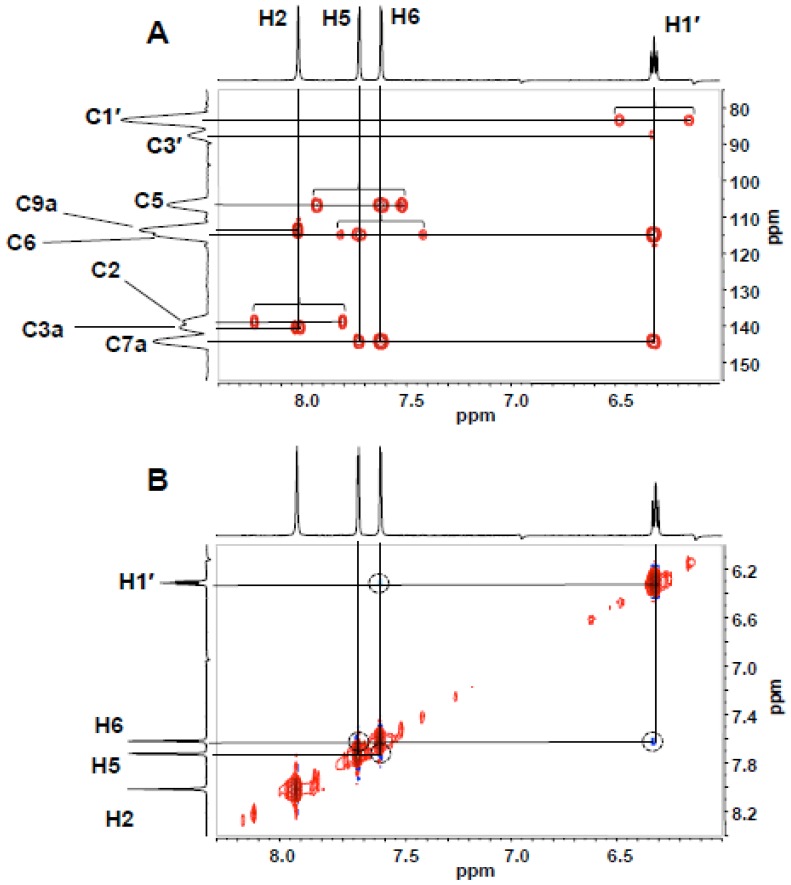
Expansions of the HMBC (A) and NOESY (B) NMR spectra (DMSO-*d_6_*) of the 12 min-eluting product of enzymatic ribosylation of 1, spanning the region of H1′-etheno interactions. Proton signals are identified on the marginal spectral traces. In the HMBC spectrum, unsuppressed one-bond C-H couplings are indicated by brackets.

**Figure 6:**
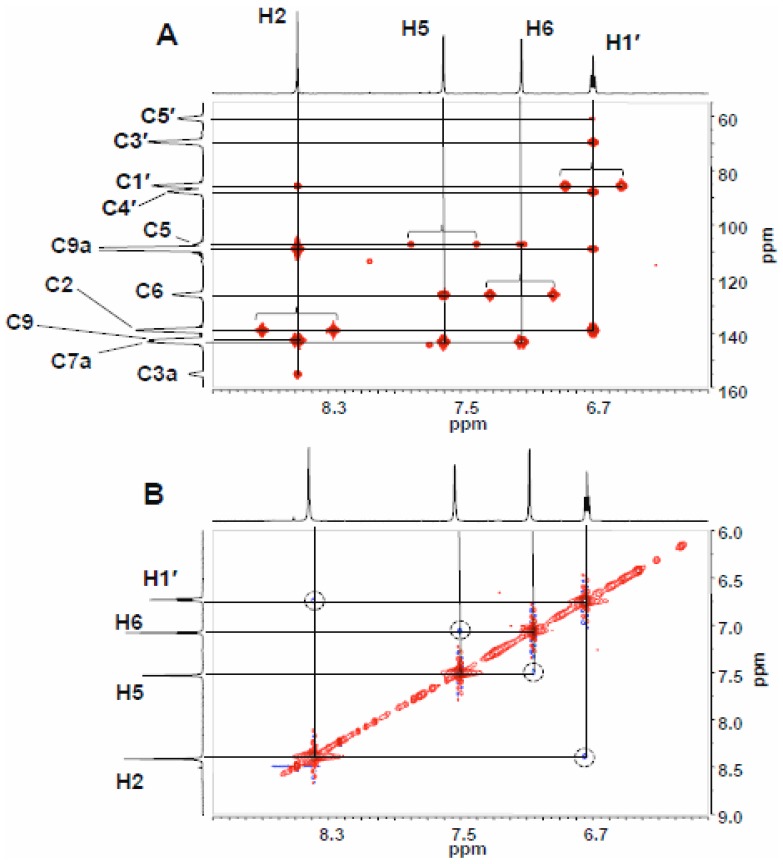
Expansions of the HMBC (A) and NOESY (B) NMR spectra (DMSO-*d_6_*) of the 16 min-eluting product of enzymatic ribosylation of 1, spanning the region of H1′-etheno interactions. Proton signals are identified on the marginal spectral traces. In the HMBC spectrum, unsuppressed one-bond C-H couplings are indicated by brackets.

The expansion of the HMBC spectrum of the product eluting at 12 min (Fig. 5A) shows coupling between H1′ and C7a and between H1′ and C6, consistent with sugar substitution at N7, while the absence of coupling between H1′ and C3a, between H1′, C9a or C2 or between H2 and C1′ is inconsistent with ribosylation at N1 or N3. N7 ribosylation is further supported by the NOESY spectrum (Fig. 5B), where a cross peak between H1′ and H6 is observed and no NOESY interactions are detected between H2 and any of the deoxyribose protons. In the HMBC spectrum of the nucleoside eluting at 16 min (Fig. 6A), ribosylation at N1 is established by H1′/C2, H2/C1′ and H1′/C9a coupling and the absence of H1′/C3a coupling. (Full HMBC and NOESY spectra are presented as S2 and S3 Figures, respectively.) A NOESY cross peak between H1′ and H2 (Fig. 6B) is the only NOESY interaction between H1′ and the base, consistent with N1 ribosylation assigned on the basis of the HMBC spectrum. (Full HMBC and NOESY spectra are presented as S4 and S5 Figures, respectively.)

**Figure 7:**
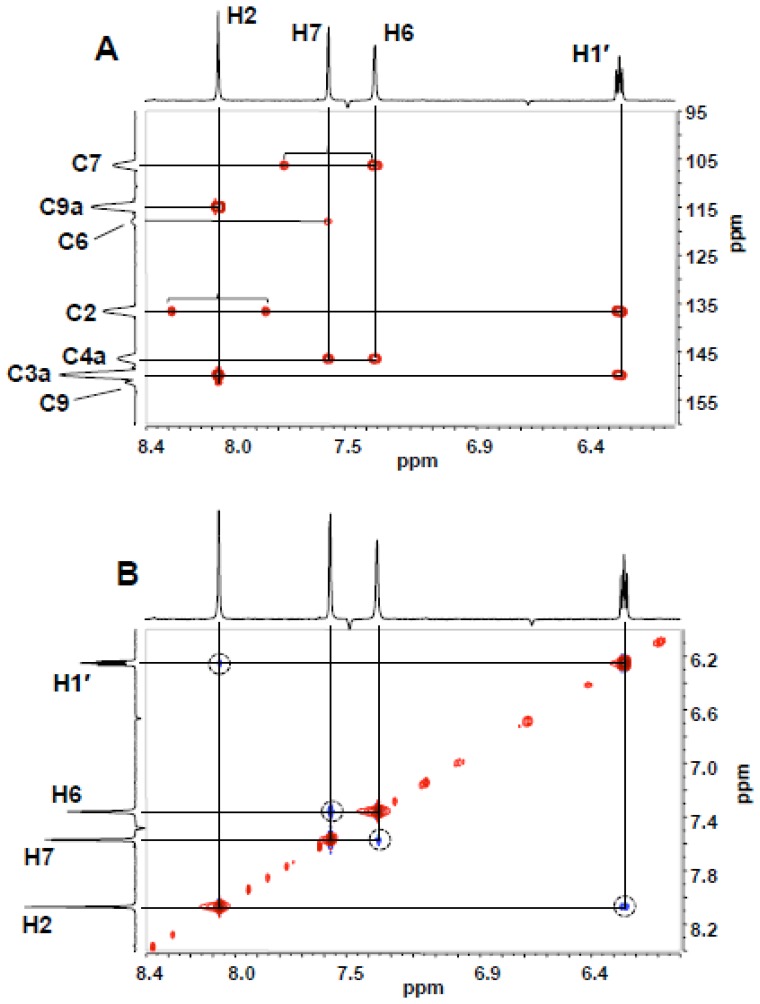
HMBC (A) and NOESY (B) NMR spectra (D_2_O) of 18 minute-eluting product of enzymatic glycosylation spanning the region of H1′-etheno interactions. Proton signals are identified on marginal traces. In the HMBC spectrum, unsuppressed 1-bond C-H couplings are identified by brackets.

As discussed above, the UV absorbance band at 290 nm indicates that the base moiety of the minor enzymatic products eluting at 18 and 22 min is the linear tricyclic 5,9-dihydro-9-oxoimidazo[1,2-*a*]purine framework. The ^1^H NMR, HMBC and NOESY spectra of the product eluting at 18 min were identical to those of an authentic sample of 5,9-dihydro-9-oxo-3-(*β*-D-2-deoxyribofuranosyl)-imidazo[1,2-*a*]purine, confirming the site of ribosylation at N3. In the HMBC spectrum (Fig. 7A), H1′/C3a and H1′/C2 cross-peaks require that the sugar be attached at N3 and consistent with this observation, the only NOESY interaction observed between the base and sugar is an H1′,H2 cross-peak (Fig. 7B). (Full HMBC and NOESY spectra are presented as S6 and S7 Figures, respectively.)

Although it was not possible to acquire 2-dimensional NMR data on the 22 min-eluting sample, the structure 5,9-dihydro-9-oxo-1-(*β*-D-2-deoxyribofuranosyl)-imidazo[1,2-*a*]purine is assigned based on a report [14] of this isomer as a minor glycosylation product of **2** by a partially purified mixture of NDT- and PDT-containing extracts of *L. helveticus*. In this report [14], the structure was unequivocally established by a nuclear Overhauser effect (NOE) difference spectrum that showed the expected H1′/H2 interaction. Unfortunately, the ^1^H NMR trace was presented without tabulated proton chemical shifts and a definitive comparison of ^1^H NMR shifts and coupling constants is not possible. Thus, our structural assignment with regard to regiochemistry of glycosylation must be regarded as tentative, based on the approximate coincidence of the proton signals (S8 Figure).

The Type II DRTs from *L. fermentum* and *L. leichmannii* glycosylated **1** with high efficiency. While overall efficiency of the glycosylation of **1** was independent of pH, the product profiles at higher pH show an increase in deoxyribosylation at N1 at the expense of the N7 isomer. Transribosylation by *L. helveticus* PDT was less efficient overall than transribosylation by the Type II transferases, and slightly more efficient at pH 7.5 than pH 8. The effect of pH on the product profile was reversed, with the N7 deoxyribosylated product increasing at the expense of the deoxyribosylation at N1. The three DRTs generated the products eluting at 18 and 22 min efficiently with high selectivity for the product eluting at 18 min regardless of pH. Although *E. coli* PNP was nearly an order of magnitude less efficient than the DRT enzymes at generating the products from **1**, all of the enzymes glycosylated **2** efficiently. Table 3 summarizes extent of conversion and product profile of **1** and Table 4 summarizes extent of conversion and product profile of **2**.

**Table 3:** Glycosylation of 8,9-dihydro-9-oxoimidazo[2,1-*b*]purine (1).

Enzyme	pH	Conversion in 17 h	Ratio of N1/N7 glycosylation
*L. helveticus* PDT	8.0	41^a^	3.3
*L. helveticus* PDT	7.5	62^a^	2.9
*L. fermentum* NDT	8.0	92^a^	0.28
*L. fermentum* NDT	7.5	85^a^	0.81
*L. leichmannii* NDT	8.0	98^a^	0.15
*L. leichmannii* NDT	7.5	93^a^	0.28
*E. coli* PNP	8.0	5^b^	0.19

a nmole/mg protein.

b nmole/unit protein.

**Table 4:** Glycosylation of 5,9-dihydro-9-oxoimidazo[1,2-*a*]purine (2).

Enzyme	pH	Conversion in 17 h	Ratio of N3/N1 glycosylation
*L. helveticus* PDT	8.0	7.5^a^	16.9
*L. helveticus* PDT	7.5	1.8^a^	15.6
*L. fermentum* NDT	8.0	2.6^a^	16.6
*L. fermentum* NDT	7.5	3.5^a^	14.6
*L. leichmannii* NDT	8.0	4.4^a^	13.6
*L. leichmannii* NDT	7.5	4.5^a^	12.6
*E. coli* PNP	8.0	4.2^b^^,^ c	19.0

a nmole/mg protein.

b nmole/unit protein.

c estimate based on complete glycosylation of substrate.

### Chemical Glycosylation

*O* ^9^-Benzyl-protected 8,9-dihydro-9-oxoimidazo[2,1-*b*]purine was glycosylated and deprotected by standard methods [19], to examine the steric accessibility of N3 (N9 in the Gua framework) to chemical deoxyribosylation in solution, which should impose less rigorous steric constraints than the active site of the enzyme. The glycosylation reaction yielded only two nucleoside products, which were identical by ^1^H NMR and NOESY spectra to the N1 and N7 deoxyribosides generated enzymatically. Thus deoxyribosylation at N3 is not favorable even in the absence of constraints imposed by the binding requirements of the active site residues.

### Synthesis of 8,9-Dihydro-9-oxo-3-(2-deoxy-β-D-ribofuranosyl)-imidazo[2,1-b]purine (3)

**Figure 8:**
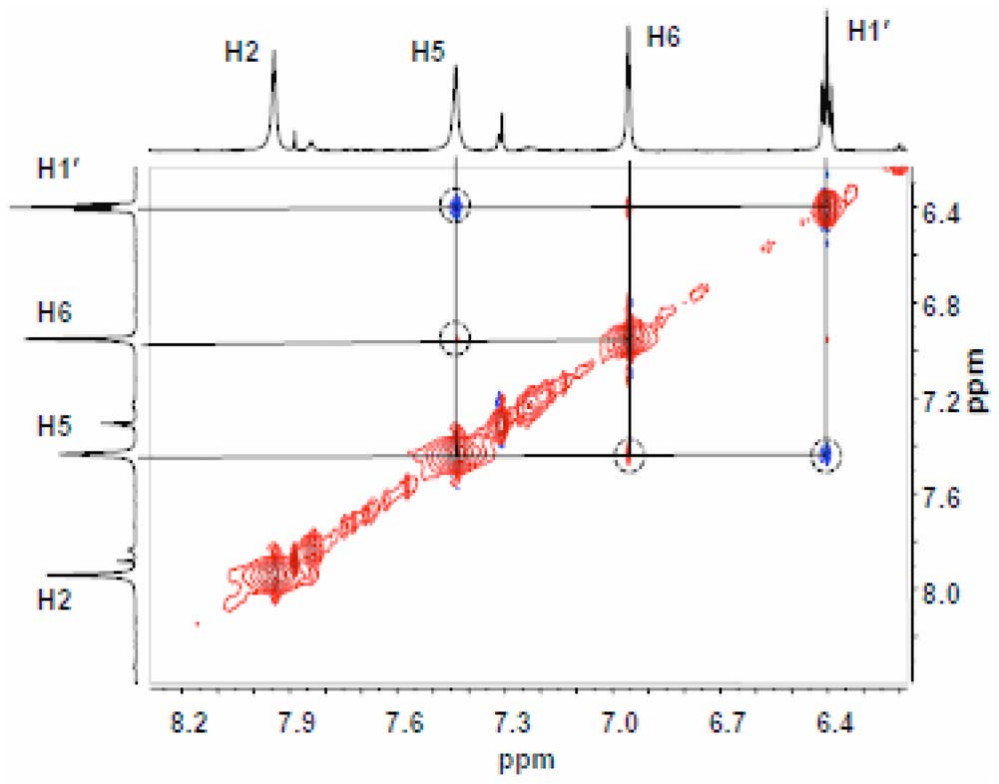
NOESY NMR spectrum (DMSO-*d_6_*) of 3 spanning the region of H1′-etheno interactions. Protons are identified on the marginal traces.

We felt that for completeness as well as for absolute confirmation of the structures assigned to the enzymatic and chemical glycosylation products of **1**, comparison with the authentic N3 glycosylated isomer obtained by an unambiguous synthetic route would be appropriate. Several syntheses of **3** starting with Guo or dGuo have been reported. The electronic absorption spectra and one-dimensional ^1^H NMR spectra provided in support of the target structure do not offer definitive means to distinguish between the isomers of ribosylation. The insensitivity of electronic spectra and the chemical shifts of the deoxyribose protons to the position of ribosylation on the periphery of the base as well as the cited variability of etheno proton chemical shifts [34] require additional characterization of the target compound by 2-dimensional NMR experiments. We employed an unambiguous synthetic route to **3** based on cycloaddition of bromoacetaldehyde to *O* ^6^-protected dGuo followed by deprotection [20], [35]. Consistent with the cited variability of etheno proton chemical shifts [35], the chemical shifts of etheno proton signals H5 and H6 of the N3-glycosylated product and the products of the two reported syntheses [20], [35] all differ, notwithstanding the fact that the ^1^H NMR spectra were recorded in the same solvent (DMSO-*d*_6_). A nuclear Overhauser effect has been reported between H1′ and H5 for *O* ^9^-protected *O* ^9^-benzyl-8,9-dihydro-9-oxo-3-(*β*-D-ribofuranosyl)-imidazo[2,1-*b*]purine [34]. However, the equivalent experiment has not been reported for the deoxy analog or the target deoxynucleoside, and consequently we recorded the NOESY spectrum of our 8,9-dihydro-9-oxo-3-(2-deoxy-*β*-D-ribofuranosyl)-imidazo[2,1-*b*]purine. The expected H1′,H5 interaction was observed (Fig. 8) but interestingly, an H1′, H2 NOESY interaction was not detected. The full NOESY spectrum is given in S9 Figure.

In the report of the synthesis of the *O* ^6^-protected nucleoside, information was not provided regarding the presence or absence of a nuclear Overhauser effect between H1′ and H2 in the structurally related *O* ^9^-benzyl nucleoside. The absence of this interaction could be explained by hindered rotation around the glycosidic bond; nevertheless, the synthetic N3-ribosyl derivative is clearly distinct from the products of enzymatic and chemical glycosylation of the imidazo[2,1-*b*]purine system. Moreover, the HPLC retention time obtained under conditions identical to those of the work-up of the enzymatic glycosylations was significantly shorter (5.4 min) than the retention times of the isomeric deoxyribosylated derivatives obtained by enzymatic or chemical synthesis.

## Discussion

**Figure 9:**
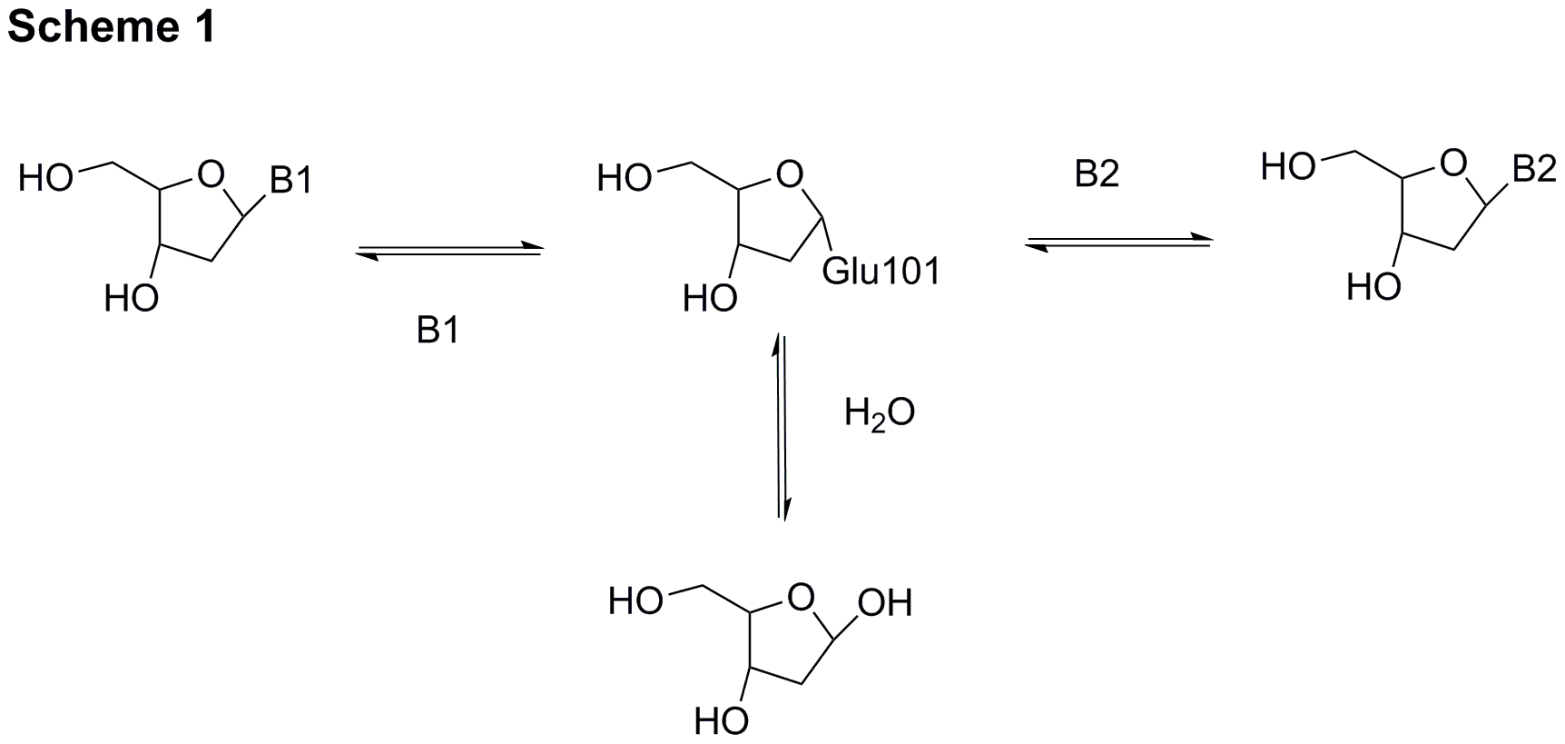
Pathway of deoxyribosyl transfer.

NDT from *L. leichmannii* [11] deoxyribosylates adenine bearing the bulky C8 substituents Br, Cl or CF_3_, at both N3 and N9 (referred to the adenine framework). Crude *L. helveticus* extracts have been reported to deoxyribosylate guanine having a 5- or 6-membered ring fused on the Watson-Crick pairing edge [14] at both N7 and N9 (referred to the guanine framework) while *E. coli* PNP similarly yields mixtures of products with purines bearing bulky *N* ^2^ substituents [12] as well as with certain base analogs [36], [37]. The deoxyribosyl transfer reaction proceeds via a ping-pong–bi-bi mechanism [38], illustrated in Fig. 9 for the *L. helveticus* enzyme. The deoxyribose at the active site of *L. helveticus* PDT is anchored by hydrogen bonding with Ser14, Tyr17, and Asp95, by a covalent α linkage of oxygen atom OE2 of Glu101 to deoxyribose C1′ and by hydrogen bonding with Asn128# from a neighboring unit [8]. An S_N_2 displacement of Glu101 yields the β-anomer of the glycosylated acceptor base thus retaining the β-anomeric configuration of the deoxynucleoside [36]. The natural base acceptor Ade is held in place by hydrogen bonds with Tyr167# (from a neighboring unit) and Asp75 and is further stabilized in the active site by π-stacking with Phe45 [9].

### Binding of 1 in Multiple Conformations

Our crystal structure at 2.1 Å resolution indicates that **1** binds in four distinct, overlapping conformations in the PDT active site. In all orientations, the planar purine base is stabilized by π-stacking with Phe45 as observed in the case of *L. helveticus* PDT bound to dAdo and other purine derivatives [8]. Thus in our structure, the purine remains in the same plane with orientations related to each other by rotation about an axis perpendicular to the plane of the ring. Binding in conformations related by rotation around the normal to the purine plane indicates that there is sufficient space in the active site to accommodate the larger tricyclic, modified base, although the PDT had been thought to be more selective than the NDT with respect to acceptor molecules.

In the first orientation (Fig. 1A) the tricyclic skeleton is positioned with N8 in the vicinity of the ribose binding site at Glu101. However, N8 is not anticipated to be strongly nucleophilic, and a corresponding glycosylation product was not identified. In the second conformation (Fig. 1B), the base is rotated by approximately −120° but does not offer any nucleophilic sites favorable for attack and hence is also predicted to be unproductive. Further rotation by −120° yields the third orientation (Fig. 1C) and places N1 near the sugar binding site, in a position suitable for glycosylation. In the fourth orientation (Fig. 1D), the base is bound in the active site in such a way that N7 is positioned near the sugar binding site and is oriented for nucleophilic attack by the deoxyribosyl moiety. Thus the crystal structure is compatible with transfer of the deoxyribosyl group from Glu101 to positions N1 and N7 of the tricyclic base as observed in the product profile.

The product profile from deoxyribosyl transfer by *L. helveticus* PDT to **1** shows a marked preference for addition at N1 relative to N7 (Table 3), which appears to be in line with the somewhat more extensive H-binding network in orientation 2C. In contrast, glycosylation of **1** by the partially purified protein isolated from *L. helveticus* was reported to attach the deoxyribose at N1 and N3 (corresponding to N7 and N9, respectively, of the guanine skeleton) in equal amounts at pH 6 and with a marked preference for N3 at pH 8 [14]. The published structural assignments [14] appear to be based on the assumption that N1 and N3 would be the target sites for deoxyribosylation, since no spectroscopic confirmation of the structures was presented. Our investigation suggests that the assignment of the N3 glycosylation product be revised. For purified *L. helveticus* PDT, neither the efficiency nor regioselectivity of the transfer was as strongly pH-dependent as reported for the partially purified enzyme mixture, with efficiency being slightly higher, rather than lower at lower pH.

### Substrate Binding in *L. leichmannii* NDT and *E. coli* PNP

In the modeled *L. leichmannii* NDT [9], the planar skeleton of **1** is stabilized in the active site by π-stacking with Phe13 in a fashion analogous to the stacking interaction observed for the oxoimidazopurine with Phe45 in *L. helveticus* PDT. Modeling indicates that the active site cavity of *L. leichmannii* NDT has sufficient space to accommodate the tricyclic skeleton allowing rotation around the normal to the molecular plane with multiple orientations possible depending on alternative hydrogen bonding interactions. The most energetically favorable orientations of **1** were stabilized by hydrogen bonding with Gln46, shown to be involved in anchoring nucleobase moieties in published crystal structures. However in contrast to several studies, Asp72 did not participate in anchoring the substrate in our model. The model predicts that N1 and N7 are optimal sites for glycosylation in agreement with the observed products, although in the case of the NDT, differences in hydrogen bonding of the base with the active site residues must alter the relative ratio of the products. The relative energies of the favored orientations are in agreement with the observed preference for glycosylation at N7 (Table 3), in contrast to *L. helveticus* PDT. *L. fermentum* NDT, like *L. helveticus* PDT and *L. leichmannii* NDT, glycosylated **1** at N1 and N7 with somewhat less selectivity for N7 than *L. leichmannii* NDT (Table 3). Structural congruence of *L. fermentum* with *L. leichmannii* NDT based on sequence homology has been suggested [21] and the similarity of the product profile to that obtained with *L. leichmannii* is in accord with this suggestion. However, since a structure of *L. fermentum* NDT is not available, no modeling study was done. Both *L. leichmannii* and *L. fermentum* NDTs were slightly more efficient overall than the PDT in deoxyribosylation, and produced N1 and N7 glycosylated bases with a preference for N7. Efficiency of the transfer was not significantly pH-dependent, although the product distribution shifted toward glycosylation at N7 at higher pH. Glycosylation of multiple positions of the tricyclic base by the DRTs in our study is in line with the report of mixtures of N3 and N9 deoxyadenosines obtained with *L. leichmanii* NDT from C8-substituted Ade depending on the steric demands of the substituent [11].

The *E. coli* PNP, like the DRTs, glycosylated **1** at N1 and N7, with the N7 product predominating, although the overall reaction was much less efficient than for the DRTs. In *E. coli* PNP, a π-stacking interaction with Phe159 plays a role in orienting the plane of a purine base in a manner similar to the role of Phe45 in *L. helveticus* PDT and Phe13 in *L. leichmannii* NDT. Position with regard to rotation about the normal to the purine plane is determined by hydrogen bonding to Asp204, shown to play a key role in both catalysis and binding of purine to residues within the active site [10]. In the model, a single conformation anchored by two hydrogen bonds with Asp204 presenting N7 for deoxyribosylation is strongly favored. A second less favorable orientation making one hydrogen bond with Asp204 would result in deoxyribosylation of N1. The profile of deoxynucleoside products obtained with **1** bears out this prediction out (Table 3). Low overall efficiency of the synthesis reaction could result either from a poor fit to the active site or to sub-optimal orientations of the nucleophilic sites available for attachment of the deoxyribosyl group.

All DRTs efficiently glycosylated **2** at N3 and at a second site tentatively identified as N1, with strong selectivity for N3 (N9 of the guanine moiety). This observation is in accord with the relative efficiency for this substrate reported [14] using the partially purified *L. helveticus* enzyme mixture. *E. coli* PNP displayed the same pattern of glycosylation of **2** as the DRTs, but in contrast to the low efficiency of the PNP reaction with **1**, efficiency was comparable to that of the DRTs. Product profiles obtained with **2** are consistent with rotation around the normal to the molecular plane of the base resulting in positioning of either N1 or N3 for accepting the deoxyribose.

A common feature of the base-binding pocket of DRTs and *E. coli* PNP is a Phe that interacts with purine acceptors by π-stacking that functions to fix the position of the molecular plane. The orientation of the acceptor base within the plane is determined by hydrogen bonding interactions with other active-site residues. In the case of **1**, the same active site residues appear to be responsible for binding natural substrates. For natural substrates, selectivity of ribosylation is high, whereas in the case of the modified acceptor base, alternative hydrogen bonding possibilities result in multiple positions with respect to rotation around the normal to the molecular plane, which in turn, present different targets for attachment of the deoxyribose. The result is a distribution of products, with relative yields determined by the proportion of acceptors occupying the rotational positions.

In the present study neither enzymatic nor chemical glycosylation of **1** yielded **3**, as conclusively demonstrated by comparison of HPLC retention times and NMR data with an authentic sample. In the case of the enzymatic synthesis, the regiochemistry of glycosylation appears to be determined by the positioning of nucleophilic sites resulting from specific hydrogen bonding schemes. The regiochemistry of chemical glycosylation is likely a result of steric hindrance of N3 by location of the nucleophilic target on the peripheral indentation resulting from the *N* ^2^,3-fusion of the etheno ring. Supporting this suggestion is the observation that synthesis of **3** by cycloaddition of bromoacetaldehyde to dGuo required blocking *O* ^6^ with a bulky protecting group to prevent exclusive formation of the 1,*N* ^2^-fusion product. Regioselectivity of deoxyribosylation was correctly predicted at non-natural sites of the guanine framework both by the crystal structure and models. This work supports modeling prior to synthetic efforts to accurately predict products as an aid to determining whether enzymatic synthesis can achieve target products. The results of this work will also contribute to understanding the capabilities of the transglycosylases to accommodate sterically demanding base analogs.

### Accession Codes

Complete structure factor data and final coordinates were deposited in the Protein Data Bank (www.rcsb.org): PDB ID code 4MEJ.

### Notes

For consistency and clarity, the numbering scheme based on the imidazopurine skeleton is used for the tricyclic bases throughout the text.

## Supporting Information

S1 Figure **HPLC trace, monitored at 260 nm, of mixture from ribosylation of 1 by** ***L. fermentum*** **NDT at pH 7.5.**
(TIF)

S2 Figure **HMBC spectrum (DMSO-** ***d_6_*** **) of 12 min peak from enzymic glycosylation products, identified as 8,9-dihydro-9-oxo-7-(** ***β*** **-D-2-deoxyribofuranosyl)-imidazo[2,1-** ***b*** **]purine.** ^1^H and ^13^C signal assignments are indicated on marginal traces. Unsuppressed 1-bond C-H couplings are indicated by brackets.
(TIF)

S3 Figure **NOESY spectrum (DMSO-** ***d_6_*** **) of 12 min peak from enzymic glycosylation products, identified as 8,9-dihydro-9-oxo-7-(** ***β*** **-D-2-deoxyribofuranosyl)-imidazo[2,1-** ***b*** **]purine.** ^1^H signal assignments are indicated on marginal traces.
(TIF)

S4 Figure **HMBC spectrum (DMSO-** ***d_6_*** **) of 16 min peak from enzymic glycosylation products, identified as 8,9-dihydro-9-oxo-1-(** ***β*** **-D-2-deoxyribofuranosyl)-imidazo[2,1-** ***b*** **]purine.** ^1^H and ^13^C signal assignments are indicated on marginal traces. Unsuppressed 1-bond C-H couplings are indicated by brackets.
(TIF)

S5 Figure **NOESY spectrum (DMSO-** ***d_6_*** **) of 16 min peak from enzymic glycosylation products, identified as 8,9-dihydro-9-oxo-1-(** ***β*** **-D-2-deoxyribofuranosyl)-imidazo[2,1-** ***b*** **]purine.** ^1^H signal assignments are indicated on marginal traces.
(TIF)

S6 Figure **HMBC spectrum (DMSO-** ***d_6_*** **) of 18 min peak from enzymic glycosylation products, identified as 5,9-dihydro-9-oxo-3-(** ***β*** **-D-2-deoxyribofuranosyl)-imidazo[1,2-** ***a*** **]purine.** ^1^H and ^13^C signal assignments are indicated on marginal traces. Unsuppressed 1-bond C-H couplings are indicated by brackets.
(TIF)

S7 Figure **NOESY spectrum (DMSO-** ***d_6_*** **) of 18 min peak from enzymic glycosylation products, identified as 5,9-dihydro-9-oxo-3-(** ***β*** **-D-2-deoxyribofuranosyl)-imidazo[1,2-** ***a*** **]purine.** ^1^H signal assignments are indicated on marginal traces.
(TIF)

S8 Figure **^1^H NMR spectrum (500 MHz, DMSO-** ***d_6_*** **) of 22 min peak from enzymic glycosylations, identified as 5,9-dihydro-9-oxo-1-(** ***β*** **-D-2-deoxyribofuranosyl)-imidazo[1,2-** ***a*** **]purine.** Peak assignments given on trace are tentative, based on Ref. (*6*).
(TIF)

S9 Figure **NOESY spectrum (DMSO-** ***d_6_*** **) of 8,9-dihydro-9-oxo-3-(** ***β*** **-D-2-deoxyribofuranosyl)-imidazo[2,1-** ***b*** **]purine.** ^1^H signal assignments are indicated on marginal traces.
(TIF)

S1 Materials **Procedures for chemical synthesis of 3 and chemical glycosylation of 1.**
(DOCX)
