# Supplementary figures and images for: Ethenoguanines Undergo Glycosylation by Nucleoside 2′-Deoxyribosyltransferases at Non-Natural Sites
Source: PLoS One. 2014 Dec 18;9(12):e115082. doi: 10.1371/journal.pone.0115082 (PMC4270796; doi:10.1371/journal.pone.0115082)

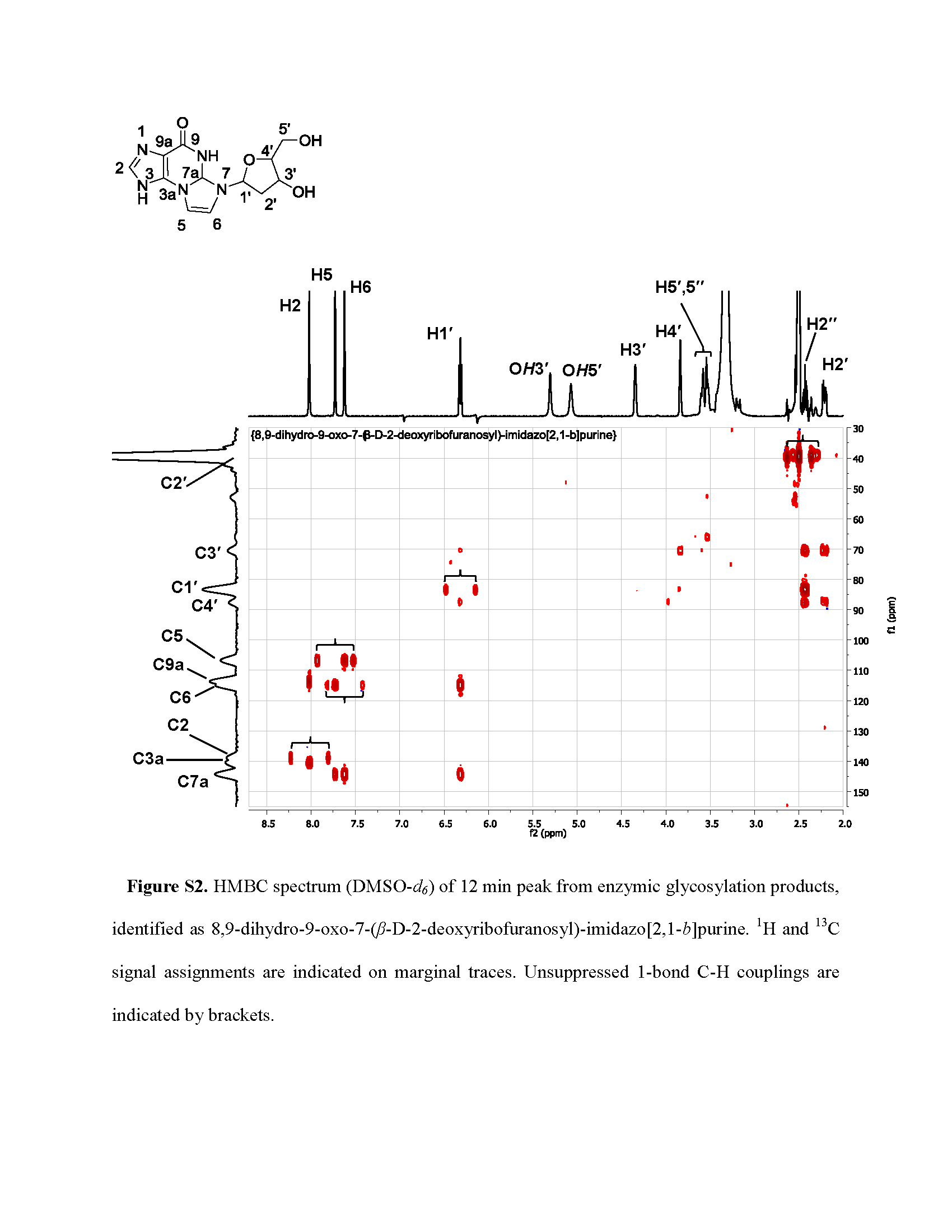

Supplement: S2 Figure — HMBC spectrum (DMSO- d6 ) of 12 min peak from enzymic glycosylation products, identified as 8,9-dihydro-9-oxo-7-( β -D-2-deoxyribofuranosyl)-imidazo[2,1- b ]purine. 1H and 13C signal assignments are indicated on marginal traces. Unsuppressed 1-bond C-H couplings are indicated by brackets. (TIF) [file pone.0115082.s002.tif]

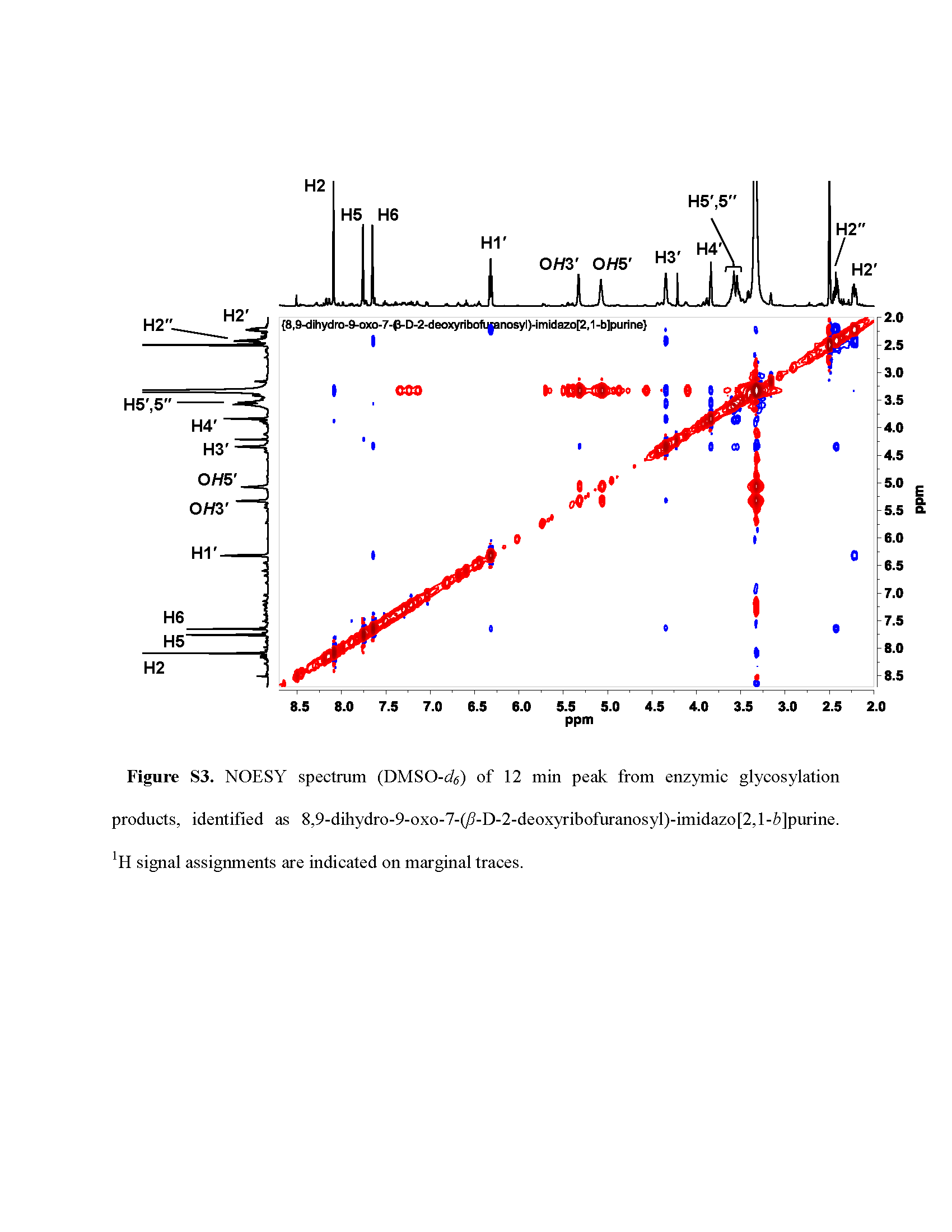

Supplement: S3 Figure — NOESY spectrum (DMSO- d6 ) of 12 min peak from enzymic glycosylation products, identified as 8,9-dihydro-9-oxo-7-( β -D-2-deoxyribofuranosyl)-imidazo[2,1- b ]purine. 1H signal assignments are indicated on marginal traces. (TIF) [file pone.0115082.s003.tif]

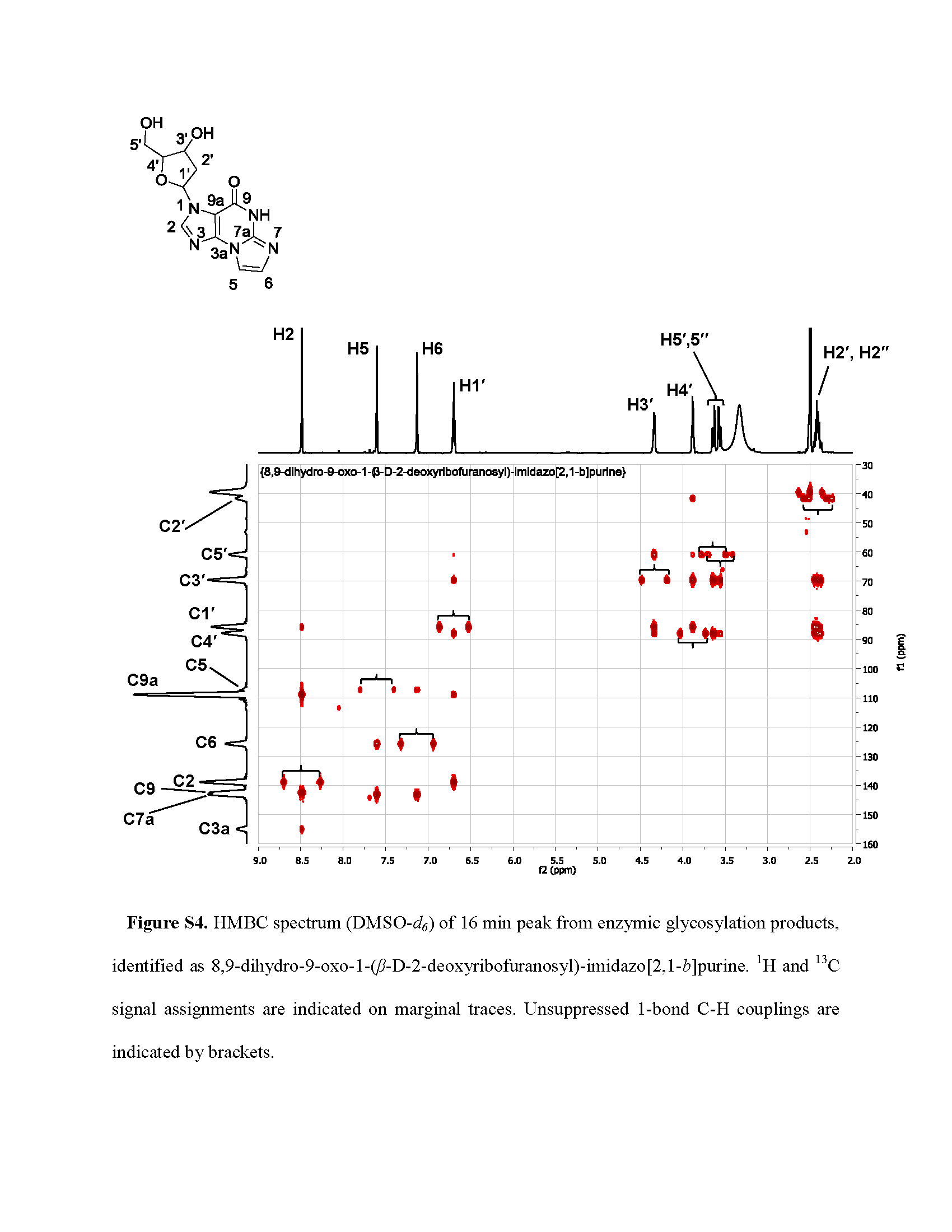

Supplement: S4 Figure — HMBC spectrum (DMSO- d6 ) of 16 min peak from enzymic glycosylation products, identified as 8,9-dihydro-9-oxo-1-( β -D-2-deoxyribofuranosyl)-imidazo[2,1- b ]purine. 1H and 13C signal assignments are indicated on marginal traces. Unsuppressed 1-bond C-H couplings are indicated by brackets. (TIF) [file pone.0115082.s004.tif]

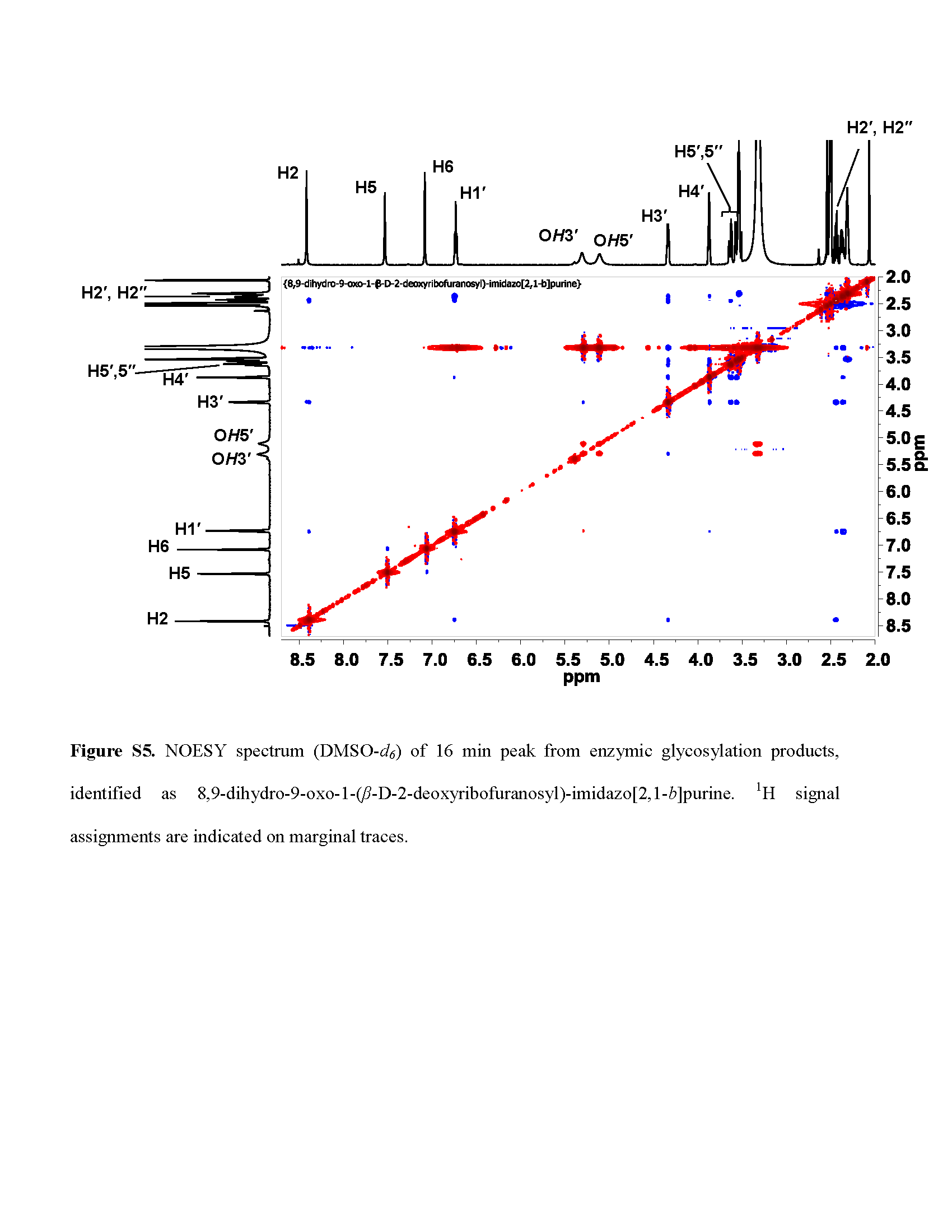

Supplement: S5 Figure — NOESY spectrum (DMSO- d6 ) of 16 min peak from enzymic glycosylation products, identified as 8,9-dihydro-9-oxo-1-( β -D-2-deoxyribofuranosyl)-imidazo[2,1- b ]purine. 1H signal assignments are indicated on marginal traces. (TIF) [file pone.0115082.s005.tif]

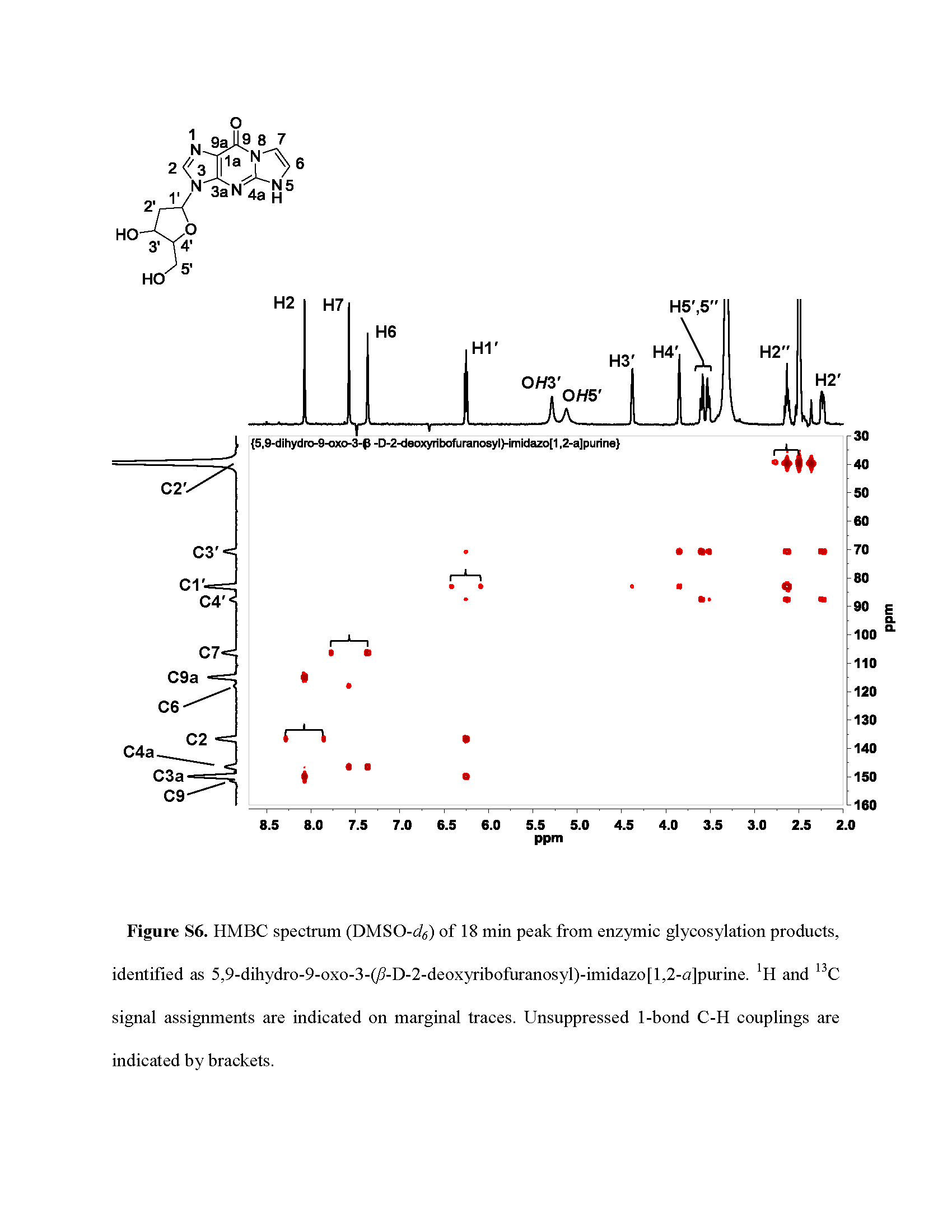

Supplement: S6 Figure — HMBC spectrum (DMSO- d6 ) of 18 min peak from enzymic glycosylation products, identified as 5,9-dihydro-9-oxo-3-( β -D-2-deoxyribofuranosyl)-imidazo[1,2- a ]purine. 1H and 13C signal assignments are indicated on marginal traces. Unsuppressed 1-bond C-H couplings are indicated by brackets. (TIF) [file pone.0115082.s006.tif]

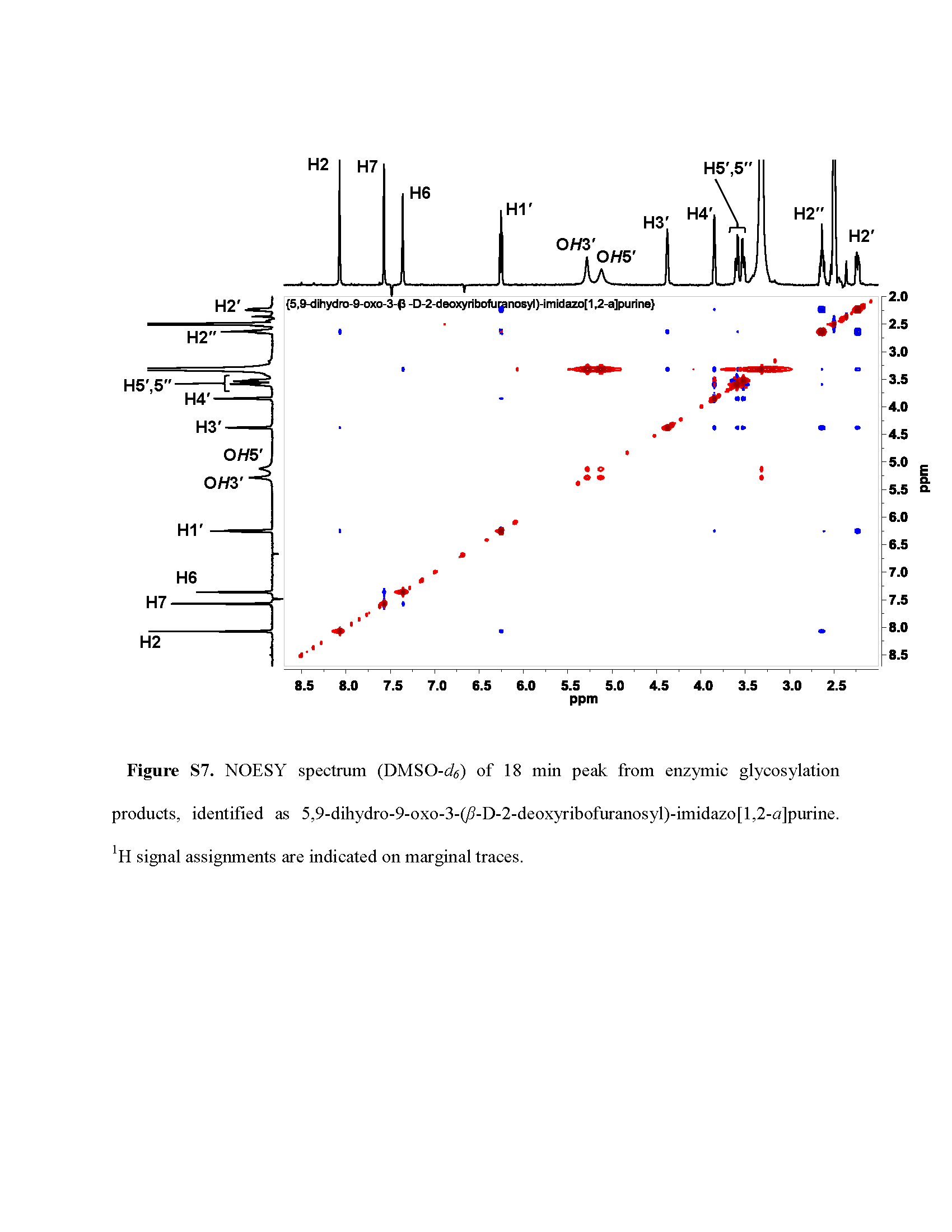

Supplement: S7 Figure — NOESY spectrum (DMSO- d6 ) of 18 min peak from enzymic glycosylation products, identified as 5,9-dihydro-9-oxo-3-( β -D-2-deoxyribofuranosyl)-imidazo[1,2- a ]purine. 1H signal assignments are indicated on marginal traces. (TIF) [file pone.0115082.s007.tif]

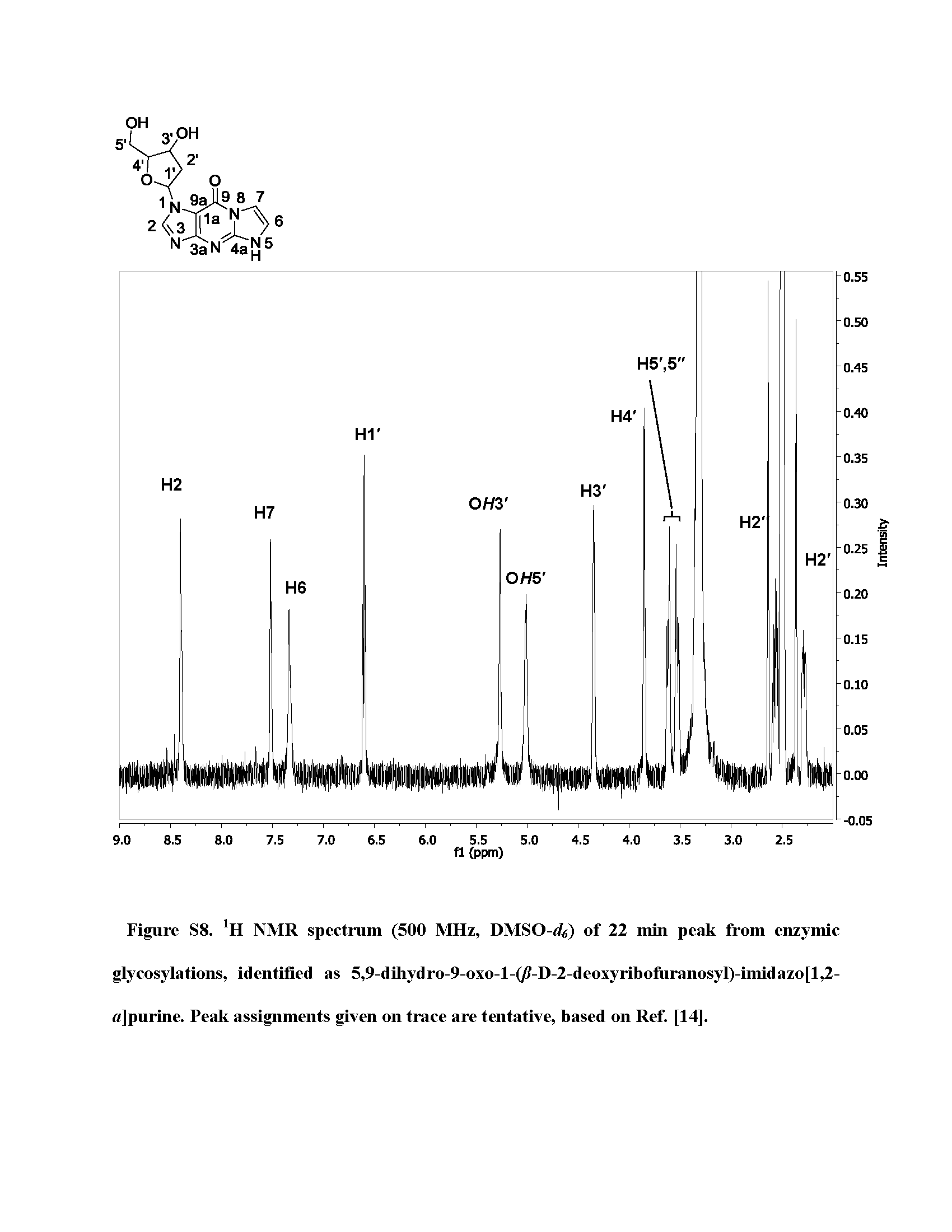

Supplement: S8 Figure — 1H NMR spectrum (500 MHz, DMSO- d6 ) of 22 min peak from enzymic glycosylations, identified as 5,9-dihydro-9-oxo-1-( β -D-2-deoxyribofuranosyl)-imidazo[1,2- a ]purine. Peak assignments given on trace are tentative, based on Ref. (6). (TIF) [file pone.0115082.s008.tif]

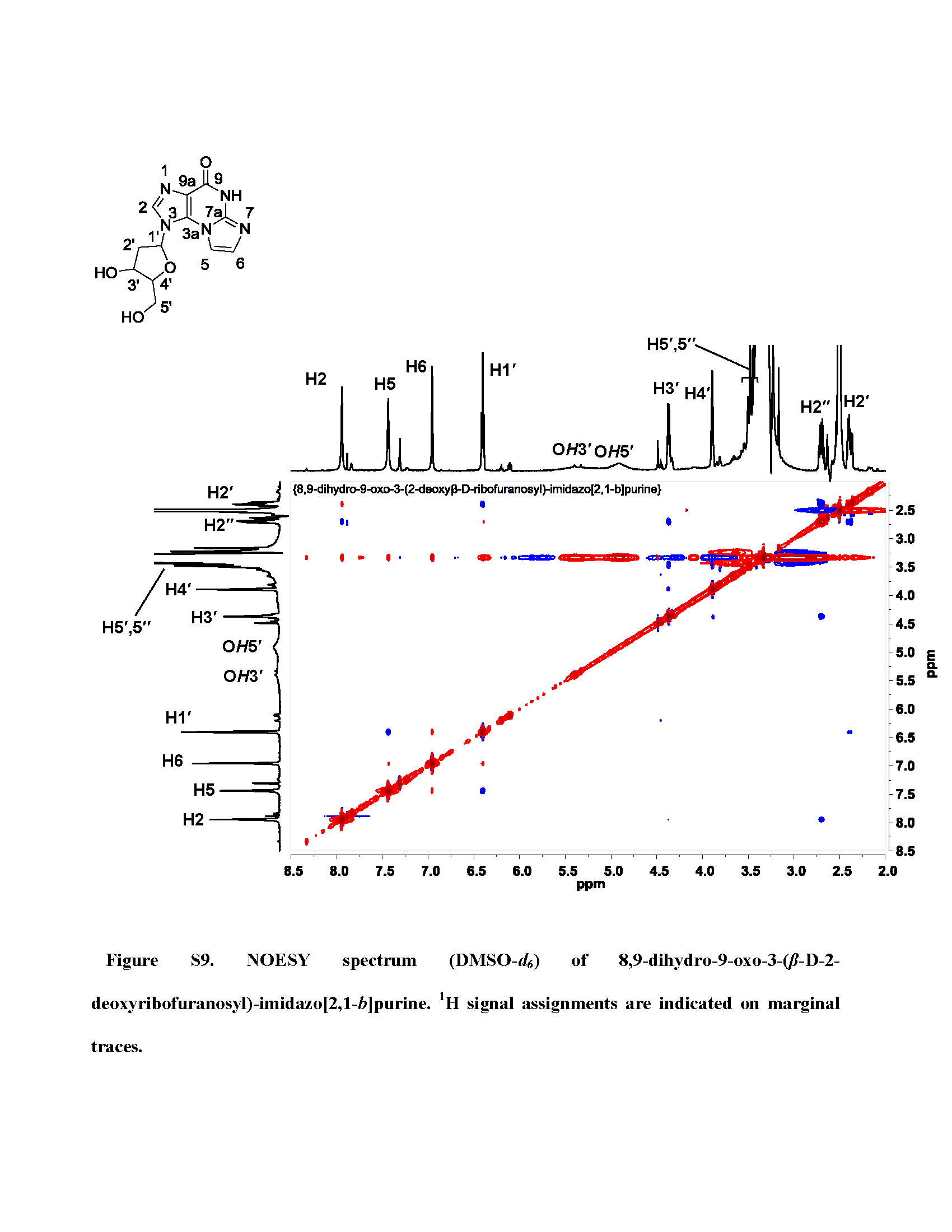

Supplement: S9 Figure — NOESY spectrum (DMSO- d6 ) of 8,9-dihydro-9-oxo-3-( β -D-2-deoxyribofuranosyl)-imidazo[2,1- b ]purine. 1H signal assignments are indicated on marginal traces. (TIF) [file pone.0115082.s009.tif]
