# Supplementary material for: Ethenoguanines Undergo Glycosylation by Nucleoside 2′-Deoxyribosyltransferases at Non-Natural Sites
Source: PLoS One. 2014 Dec 18;9(12):e115082. doi: 10.1371/journal.pone.0115082 (PMC4270796; doi:10.1371/journal.pone.0115082)
Supplement: S1 Materials — Procedures for chemical synthesis of 3 and chemical glycosylation of 1. (DOCX) [file pone.0115082.s010.docx]

Ethenoguanines undergo glycosylation by nucleoside 2´-deoxyribosyltransferases at non-natural sites

Wenjie Ye^‡^, Debamita Paul ^§^, Lina Gao^‡^, Jolita Seckute^§^, Ramiah Sangaiah^‡|^, Karupiah Jayaraj^‡^, Zhenfa Zhang^‡^, Pierre A. Kaminski ^[
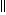
](http://pubs.acs.org/doi/abs/10.1021/bi962438a#bi962438aAF5)^, Steven E. Ealick*^,^ ^§^, Avram Gold*^,‡^, Louise M. Ball^¥,‡^

‡ The University of North Carolina at Chapel Hill

^§^ Cornell University

^[
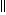
](http://pubs.acs.org/doi/abs/10.1021/bi962438a#bi962438aAF5)^ Institut Pasteur

^|^ Deceased

**SUPPORTING INFORMATION**

**MATERIALS S1.** Procedures for chemical synthesis of **3** and chemical glycosylation of **1.**

***Chemical synthesis of 8,9-dihydro-9-oxo-3-(2-deoxy-β-D-ribofuranosyl)-imidazo[2,1-b]purine (3).*** 3′,5′-Di-*O*-acetyl-2′-deoxyguanosine*.* 3′,5′-Di-*O*-acetyl-2′-deoxyguanosine was synthesized by slight modification of a published procedure.^1^ dGuo (267 mg, 1 mM) and tetraethylammonium bromide (770 mg) were dissolved in distilled water and then lyophilized. The residue was stirred with acetic anhydride in anhydrous pyridine for 18 h protected from light. Excess acetic anhydride was destroyed by addition of 5 mL ethanol, the solvents removed under vacuum and 3′,5′-di-*O*-acetyl-2′-deoxyguanosine was purified by crystallization from methanol (330mg, 95%). UV-vis (MeOH) λmax 251 nm; positive ESI-MS *m/z* (rel intensity) 725 (100) [M_2_+Na]^+^, 703 (50) [M_2_+H]^+^, 352 (28) [M+H]+,152 (42) [M+H–deoxyribose]+. 1H NMR (methanol-*d4*) 7.90 (s, 1H), 6.30 (ψt, 1H, *J* = 6.6 Hz ), 5.47 (m,1H), 4.35-4.44 ( m, 2H), 4.33 (m, 1H), 3.02(m, 1H), 2.59(ddd, 1H, *J* = 14.5, 6.2, 2.5 Hz), 2.15 (s, 3H), 2.09 (s, 3H) ppm.

*O*6-Benzyl-3′,5′-di-*O*-acetyl-2′-deoxyguanosine*.*  To a suspension of 3′,5′-di-*O*-acetyl-2′-deoxyguanosine (200 mg) under Ar in dry tetrahydrofuran (8 mL), were added triphenylphosphine (194.8 mg) and benzyl alcohol (102.4 μL).  Diisopropyl azodicarboxylate (65.2 mg) was added drop-wise with stirring in an ultrasonic bath at 25 °C. After 48 hr, the reaction solution was evaporated under reduced pressure. Semipreparative TLC on SiO2 eluted with 3: 97 (v/v) CH3OH/CH2Cl2, yielded crystalline of *O*6-benzyl-3′,5′-di-*O*-acetyl-2′-deoxyguanosine (35 mg, 14%, *R*f 0.31). Positive ESI-MS *m/z* (rel intensity) 464 (100) [M+Na]+, 442 (58) [M+H]+, 242 (94) [M+H–deoxyribose]+. 1H NMR (methanol-*d4*) 7.88 (s, 1H), 7.31 (t, 2H, *J* = 7.4 Hz), 7.24 (m, 3H), 6.27 (dd, *J* = 7.5, 6.6 Hz 1H), 5.44 (m, 1H), 5.31 (s, 2H), 4.39 (dd, 1H, *J* =11.7, 4.5 Hz), 4.33 (dd, 1H, *J* = 11.7, 4.5 Hz), 4.29 (m, 1H), 3.03 (m, 1H), δ 2.55 (ddd, 1H, *J* = 14.2, 6.6, 2.6, Hz) 2.10 (s, 3H), 2.03 (s, 3H) ppm.

*O*6-Benzyl-2′-deoxyguanosine. *O*6-Benzyl-3′,5′-di-*O*-acetyl-2′-deoxyguanosine (20 mg) was dissolved in  2M methanolic ammonium hydroxide (8 mL) and stirred overnight at room temperature. Following the evaporation of solvent, the residue was purified by semipreparative TLC on SiO2 eluted with 15: 85 (v/v) CH3OH/CH2Cl2 to give *O*6-benzyl -2′-deoxyguanosine (15.9mg, 98%, *R*f 0.6). UV (MeOH) λmax 256 nm. Positive ESI-MS *m/z* (rel intensity) 380 (100) [M+Na]+, 358 (8) [M+H]+. 1H NMR (methanol-*d4*) 7.98 (s, 1H), 7.31 (ψt, 2H, *J* = 7.4 Hz), 7.25 (t, 1H, *J* = 7.4 Hz), 7.22 (d, 2H, *J* = 7.3 Hz), 6.28 (ψt, 1H, *J* = 7.0 Hz), 5.32 (s, 2H), 4.52 (td, 1H, *J* = 5.9, 2.9 Hz), 3.99 (dd, 1H, *J* = 6.7, 3.5 Hz), 3.79 (dd, 1H, *J* = 12.0, 3.6 Hz), 3.72 (dd, 1H, *J* = 12.0, 3.6 Hz), 2.71 (ddd, 1H, *J* = 13.5, 7.2, 6.2 Hz), 2.35 (ddd, 1H, *J* = 13.5, 6.9, 3.1 Hz) ppm.

*O*9-benzyl-8,9-dihydro-9-oxo-3-(2-deoxy-β-D-ribofuranosyl)-imidazo[2,1-*b*]purine. *O*6-Benzyldeoxyguanosine (18.0 mg) was dissolved in saturated aqueous NaHCO3 (6 mL) and bromoacetaldehyde diethyl acetal (1 mL, ~1.3 M) and ethanol (2 mL) were added at room temperature. The mixture was stirred at 50 ^o^C for 48 h, lyophilized and the residue purified by HPLC, eluting with the gradient program: 40% to 70% MeOH in water over 15 min, to give *O*^9^-benzyl-8,9-dihydro-9-oxo-3-(2-deoxy-*β*-D-ribofuranosyl)-imidazo[2,1-*b*]purine (0.6 mg, 3.1 %, retention time 12 min). Positive ESI-MS *m/z* (rel intensity) 382 (100) [M+H]+, 266 (41) [M+H–deoxyribose]+. Unreacted starting material (retention time 10.5 min) was collected for repetition of the cycloaddition reaction to accumulate 2.2 mg of the intermediate, which was used directly in the next reaction.

8,9-Dihydro-9-oxo-3-(2-deoxy-β-D-ribofuranosyl)-imidazo[2,1-*b*]purine (**3**)*.* *O*9-Benzyl-8,9-dihydro-9-oxo-3-(2-deoxy-*β*-D-ribofuranosyl)-imidazo[2,1-*b*]purine **(**2.2 mg) was dissolved in a mixture of conc NH4OH (0.5 mL), D.I. water (1 mL), ethanol (2.5 mL) and methanol (1 mL) and stirred over 2.2 mg 10% Pd/C catalyst (containing 50% water) under 1.1 atm H2 at 25 °C for 30 h. The reaction was monitored by HPLC using a gradient program: 25% to 30% MeOH in H2O over 5min, then 30% to 90% MeOH in H2O over the next 9 min. Material eluting at a retn time of 5.4 min was collected. Volatiles were removed under a stream of Ar, and the remaining aqueous solution lyophilized. The residue was purified by HPLC, using the above gradient program to give 8,9-dihydro-9-oxo-3-(2-deoxy-*β*-D-ribofuranosyl)-imidazo[2,1-*b*]purine (1.6 mg, 95% yield). Over all yield based on *O*6-benzyl-dGuo consumed was estimated to be 6 %. UV λmax (MeOH) 228, 263 nm. Positive ESI-MS *m/z* (rel intensity) 314 (100) [M+Na]+, 292 (57) [M+H]+,176 (32) [M+H–deoxyribose]+. Positive ESI-MS/MS *m/z* 292 [M+H]+, 176 [M+H–deoxyribose]+. 1H NMR (DMSO-*d6*) 7.94 (s, 1H, H2), 7.44 (bs, 1H, H5), 6.96 (bs, 1H, H6), 6.40 (t, 1H, *J* = 6.2 Hz, H1′), 4.26 (dd, 1H, *J* = 10.1, 4.4 Hz, H3′), 3.89 (q, 1H, , *J* = 4.4 Hz,  H4′), 3.49 (dd, 1H, *J* = 11.8, 4.4 Hz, H5′ or H5′′), 3.43 (dd, 1H, partially overlapping H2O, H5′ or H5′′), 2.70 (dt, 1H, *J* = 12.9, 6.2 Hz, H2''), 2.39 (m, 1H, H2') ppm (NOESY spectrum with marginal ^1^H traces, Figure S8).

*Synthesis of 8,9-dihydro-9-oxoimidazo[2,1-b]purine (1).*

8,9-Dihydro-9-oxoimidazo[2,1*-b*]purine was prepared from *O*6-benzylguanine^2^ via *O*9-benzyl-8,9-dihydro-9-oxoimidazo[2,1-*b*]purine (*O*6-benzyl-*N*2,3-εGua), by a published method.^3^ UV (H2O, pH7) λmax 273, 251 nm; positive ESI-MS *m/z* 176 ([M+H]+, 83), *m/z* 351 ([M_2_+H]+, 100); 1H NMR (DMSO-*d6*) δ 8.15 (s, 1H, H2), 7.61 (d, *J* = 1.5 Hz, 1H, H5), 7.11 (d, *J* = 1.5 Hz, 1H, H6).

Two steps in the above procedures proved to be problematic. While a 91 % yield for benzylation of Gua in the synthesis of **1** has been reported, the relatively small scale benzylation of 3′,5′-di-*O*-acetyl-dGuo using this procedure gave the desired *O*^6^-benzyl-protected derivative in only 14 % yield. The yield can undoubtedly be improved, but for this work, we did not attempt to optimize conditions. As reported, the penultimate cycloaddition step in the synthesis of **3** also proved to be problematic. The basic conditions required to avoid deglycosylation of the product resulted in low conversion (3.1 %) of the protected deoxyguanosine. However, unreacted *O*^6^-benzyldeoxyguanosine could be recovered in high yield and recycled. While we did not continue the recycling process until *O*^6^-benzyldeoxyguanosine was completely consumed, nor make a systematic effort to optimize the cycloaddition conditions, it is likely that this step could be improved, increasing the overall yield.

***Chemical Glycosylation of 8,9-dihydro-9-oxoimidazo[2,1-b]purine (N2,3-ethenoguanine; 1)***

To a stirred suspension of 75 mg of *O*9-benzyl-8,9-dihydro-9-oxoimidazo[2,1-*b*]purine in 10 mL of acetonitrile (distilled from P2O5), 20 mg NaH was added and stirring continued for 2 h at ambient temperature. The reaction was then cooled in ice for 10 min and 120 mg 3,5-di-*O*-(*p*-toluyl)-2-deoxy-D-ribofuranosyl chloride added in one portion. Stirring was continued overnight, allowing the reaction to warm to ambient temperature. The reaction mixture was filtered from solid, the filter washed with ether and the combined filtrate and washings taken to dryness under vacuum. The oil was purified by semi-preparative TLC on SiO2 eluted with 6 % methanol in chloroform. Bands were collected with *R*f values 0.44 (F1, 30 mg, 17 %) and 0.20 (F2, 28 mg, 16 %). F1 1NMR (chloroform-*d*) 8.15 (s, 1H), 7.82 (q, 4H, *J* = 5.83 Hz), 7.78 (d, 1H, *J* = 1.4 Hz), 7.56 (d, 1H, *J* = 1.4 Hz), 7.48 (d, 2H, *J* = 7.8 Hz), 7.44 – 7.29 (m, overlapping with chloroform-*d*), 7.17 (d, 2H, *J* = 7.8 Hz), 6.63 (dd, 1H, *J* = 7.3, 6.3 Hz), 5.70 (d, 1H, *J* = 12.0 Hz), 5.62 (d, 1H, *J* = 12.0 Hz), 5.58 (m, 1H), 4.70 (m, 1H), 4.68 – 4.63 (m, 2H), 2.83 (ddd, 1H, *J* = 14.1, 6.3, 1.9 Hz), 2.53 (m, 1H), 2.47 (s, 3H), 2.36 (s, 3H) ppm. F2 1NMR (chloroform-*d*) 8.05 (s, 1H), 8.01 (d, 2H, *J* = 8.11 Hz), 7.81 (d, 2H, *J* = 8.11 Hz), 7.74 (d, 1H, *J* = 2.3 Hz), 7.53 (d, 2H, *J* = 7.7 Hz), 7.41 (d, 1H, *J* = 2.3 Hz), 7.33 – 7.26 (m, 5H), 7.16 (d, 2H, *J* = 7.7 Hz), 6.64 (dd, 1H, *J* = 7.4, 6.0 Hz), 5.78 – 5.70 (m, 3H), 4.79 (dd, *J* = 12.1, 3.3), 4.69 (m, 1H), 4.64 (dd, 1H, *J* = 12.1, 3.3), 2.84 (ddd, 1H, *J* = 14.2, 6.0, 2.2 Hz), 2.71 (m, 1H), 2.44 (s, 3H), 2.36 (s, 3H) ppm.

3',5'-Deprotection of glycosylated F1 and F2. F1 and F2 were each stirred in ammonia-saturated methanol (15 mL) overnight at ambient temperature. The solvent was evaporated under a stream of Ar, the resulting solids triturated with methylene chloride, filtered and the filters washed with methylene chloride. Evaporation of the collected filtrates yielded F1-1 (12.6 mg, 68 %) and F2-1 (13.0 mg, 70 %). F1-2 1NMR (DMSO-*d6*) 8.68 (s, 1H), 7.94 (d, *J* = 0.8 Hz, 1H), 7.56 (d, 2H, *J*= 7.37 Hz), 7.49 (d, 1H, *J* =  0.8 Hz), 7.41 (ψt, 2H, *J* = 7.4 Hz), 7.35 (t, 1H *J* = 7.3 Hz), 6.55 (t, 1H, *J* = 5.8 Hz), 5.63 (q, 2H, *J* = 13.0 Hz), 5.32 (d, 1H, *J* = 4.4 Hz), 5.07 (t, 1H, J = 5.2 Hz), 4.31 (dd, 1H, J = 10.2, 5.2), 3.87 (q, 1H, J = 3.9), 3.62 (dd, 1H, *J* = 11.9, 4.0 Hz), 3.54 (dd, 1H, *J* = 11.9, 4.0 Hz), 2.52 (m, overlap with DMSO-*d6*), 2.36 (m, 1H) ppm. F2-1 1NMR (DMSO-*d6*) 8.19 (d, *J* = 1.8 Hz), 8.12 (d, 1H, *J* = 1.8 Hz), 7.87 (bs, 1H), 7.58 (d, 2H, *J* = 7.4 Hz), 7.43 (ψt, 1H, *J* = ~7.2 Hz), 7.38 (t, 2H, *J* = 7.4 Hz) 6.62 (t, 1H, *J* = 6.4 Hz), 5.71 (s, 2H), 5.43 (d, 1H, *J* = 4.2 Hz), 5.07 (t, 1H, *J* = 5.1 Hz), 4.43 (m, 1H), 3.92 (dd, 1H, *J* = 7.1, 3.8 Hz), 3.68-3.55 (m, 2H), 2.56 (m, 1H), 2.39 (m, 1H) ppm.

Hydrogenolysis of *O*9-benzyl-F1-1 and -F2-1*.* *O*9-Benzyl-protected F1-1 or F2-1 (10 mg) was dissolved in methanol (6 mL) and stirred for 2 h with 2 mg 10 % Pd/C under an atmosphere of H2. The reaction mixture was filtered, the filter washed with additional methanol (5 mL) and the combined washings and filtrate taken to dryness under a stream of Ar followed by evacuation under oil pump vacuum. 16 MIN F1-2, 8,9-dihydro-9-oxo-1-(*β*-D-2-deoxyribofuranosyl)-imidazo[2,1-*b*]purine (6.7 mg, 88 %), UV-vis (H2O): λmax (ε) 217 (20867), 263 (9066) nm; positive ESI-MS *m/z* (rel intensity) 176 (50) ([M+H-deoxyribose]+, 292 (18) ( [M+H]+, 314 (20) ([M+Na]+, 330 (100) ([M+K]+; positive ESI-MS/MS *m/z* 292 [M+H]+, 176 [M+H–deoxyribose]+; ^1^H NMR (DMSO-*d6*) (Figure S1, S2) 12.38 (bs, 1H, N*H*8), 8.51 (s, 1H, H2), 7.63 (d, 1H, *J* = 1.6 Hz, H5), 7.15 (d, 1H, *J* = 1.6 Hz, H6), 6.68 (t, 1H, *J* = 6.2 Hz, H1'), 5.35 (bs, 1H, 3'-OH), 5.07 (bt, 1H, 5'-OH), 4.33 (m, 1H, H3'), 3.89 (q, 1H, J = 4.0 Hz, H4'), 3.58 (m, 2H, H5', H5''), 2.42 (m, 2H, H2'', H2') ppm. ^13^C NMR (125 MHz) 154.9 (C9), 143.0 (C7a), 142.3 (C3a), 138.6 (^1^*J*_C-H_ = 214.9 Hz, C2), 125.7 (^1^*J*_C-H_ = 190.8 Hz, C6), 108.8 (C9a), 107.1 (^1^*J*_C-H_ = 201.8 Hz, C5), 87.8 (^1^*J*_C-H_ = 147.3 Hz, C4'), 85.5 (^1^*J*_C-H_ = 170.2 Hz, C1'), 69.3 (^1^*J*_C-H_ = 148.5 Hz, C3'), 60.6 (^1^*J*_C-H_ = 147.3, 141.0 Hz, C5'), 41.3 (^1^*J*_C-H_ = 131.7 Hz, C2') ppm. 12MIN F2-2, 8,9-dihydro-9-oxo-7-(*β*-D-2-deoxyribofuranosyl)-imidazo[2,1-*b*]purine (6.0 mg, 79 %): UV-vis (H2O): λmax (ε) 218 (22342), 263 (11056) nm; ^1^H NMR (DMSO-*d6*) (Figure S3,S4) 13.52 (bs, 1H, N*H*3), 8.09 (s, 1H, H2), 7.76 (d, 1H, *J* = 2.7 Hz, H5), 7.65 (d, 1H, *J* = 2.7 Hz, H6), 6.32 (ψt, 1H, *J* = 6.8 Hz, H1'), 5.33 (d, 1H, *J* = 3.7 Hz, 3'-OH), 5.08 (bt, 1H, 5'-OH), 4.35 (m, 1H, H3’), 3.84 (dd, 1H, *J* = 6.9, 4.0 Hz, H4'), 3.56 (m, 2H, H5', H5''), 2.43 (m, 1H, H2''), 2.22 (ddd, 1H, *J* =  13.2, 6.8, 3.1 Hz, H2') ppm. ^13^C NMR (125 MHz) 144.7 (C7a), 140.6 (C3a), 139.1 (^1^*J*_C-H_ = 202.9 Hz, C2), 115.2 (^1^*J*_C-H_ = 198.5 Hz, C6), 114.0 (C9a), 106.9 (^1^*J*_C-H_ = 206.0 Hz, C5), 87.8 (C4'), 83.4 (^1^*J*_C-H_ = 165.8 Hz, C1'), 70.7 (C3'), 39.16 (^1^*J*_C-H_ = 130.7 Hz, C2') ppm.

18 Min HPLC fraction from enzymic glycosylations. Tabulated ^1^H and ^13^C shifts of 18 min fraction from enzymic glycosylations, identified as 5,9-dihydro-9-oxo-3-(*β*-D-2-deoxyribofuranosyl)-imidazo[1,2-*a*]purine (**2**). ^1^H NMR (DMSO-*d6*) 8.07 (s, 1H, H2), 7.57 (d, 1H, *J* = 2.0 Hz, H5), 7.36 (d, 1H, *J* = 2.0 Hz, H6), 6.25 (dd, 1H, *J* = 7.6, 6.2 Hz, H1′), 5.29 (bs, 1H, OH3′), 5.13 (bs, 1H, OH5′), 4.38 (m, 1H, H3′), 3.85 (m, 1H, H4′), 3.60 (dd, 1H, *J* = 11.6, 4.4 Hz, H5′ or H5′′), 3.52 (dd, 1H, J = 11.6, 4.1 Hz, H5′′ or H5′), 2.64 (ddd, *J* = 13.3, 7.61, 6.1 Hz, H, H2''), 2.23 (ddd, 1H, *J* = 13.3, 6.1, 3.0 Hz, H2') ppm. ^13^C NMR (125 MHz) 151.6 (C9), 150.1 (C3a), 146.7 (C4a), 136.7 (^1^*J*_C-H_ = 213.7 Hz, C2), 118.2 (C7), 115.1 (C9a), 106.5 (^1^*J*_C-H_ = 199.1 Hz, C6), 87.6 (^1^*J*_C-H_ = 149.6 Hz, C4'), 83.1 (^1^*J*_C-H_ = 166.7 Hz, C1'), 70.8 (C3'), 39.3 (^1^*J*_C-H_ = 126.1 Hz, C2') ppm.

22 Min HPLC fraction from enzymic glycosylations. ^1^H NMR (DMSO-*d6*) 8.40 (s, 1H), 7.51 (bs, 1H), 7.34 (bs, 1H), 6.60 (ψt, 1H, *J* = 6.7 Hz), 5.27 (bs, 1H, OH3′), 5.01 (bs, 1H), 4.34 (m, 1H), 3.85 (m, 1H), 3.62 (dt, 1H, *J* = 11.9, 4.2 Hz), 3.53 (m, 1H), 2.56 (m, 1H), 2.29 (m, 1H) ppm.

**Citations**

1. J.-L. Ravanatt, J. Cadet, Reaction of Singlet Oxygen with 2’-Deoxyguanosine and DNA. Isolation and Characterization of the Main Oxidation Products, *Chem. Res. Toxicol.* **1995**, *8*, 379 – 388.

2. Barth, C., Seitz, O., and Kunz, H. Synthesis of *6-O*-benzyl guanine and its conjugations with linkers. *Z. Naturforsch.* **2004**, *59b,* 802-806.

3. Sattsangi, P. D., Leonard N. J., and Frihart, C. R. 1,*N*^2^-Ethenoguanine and *N*^2^,3-ethenoguanine, synthesis and comparison of the electronic spectral properties of these linear and angular triheterocycles related to the Y bases, *J. Org. Chem.* **1977**, *42*, 3292 – 3296.
